# Supplementary material for: Investigation of Peptide Toxin Diversity in Ribbon Worms (Nemertea) Using a Transcriptomic Approach
Source: Toxins (Basel). 2022 Aug 8;14(8):542. doi: 10.3390/toxins14080542 (PMC9415933; doi:10.3390/toxins14080542)
Supplement: Supplementary file 1 [file toxins-14-00542-s001.zip › Supplementary File S1. Nemertean putative toxin transcripts.pdf]

toxins

Supplementary Materials: Investigation of Peptide Toxins Diversity in Ribbon Worms (Nemertea) Using Transcriptomic Approach

Anna E. Vlasenko, Vasiliy G. Kuznetsov and Timur Yu. Magarlamov*

Table S1. Toxin transcripts of *Carinoma hamanako*.

Protein family	Transcript ID	Uniprot accession	Description	e-value	Expression, TPM
			Neurotoxins		
Neurotoxin 20 family	Gene.29146_r_16340_g.29146_m.29146	TXCA_CAEEX	U3-aranetoxin-Ce1a OS=Caerostris extrusa OX=172846 PE=2 SV=1	7.4E-06	39.36
			Pore-forming toxins		
Actinoporin family. Conoidea subfamily	Gene.23587_r_11081_g.23587_m.23587	ACTP_CONCN	Conoporin-Cn1 OS=Conus consors OX=101297 PE=1 SV=1	5.1E-09	7.21
			Enzymes		
5'- nucleotidase family	Gene.35055_r_23242_g.35055_m.35055	V5NTD_NAJAT	Snake venom 5'-nucleotidase (Fragment) OS=Naja atra OX=8656 PE=1 SV=1	1.5E-46	2.84
Arthropod phospholipase D family. Class II subfamily	Gene.22051_r_9780_g.22051_m.22051	B1R2_LOXSN	Dermonecrotic toxin LspiSicTox-betaIE1ii (Fragment) OS=Loxosceles spinulosa OX=571532 PE=2 SV=1	1.2E-40	45.45
Dnase II family	Gene.12683_r_4081_g.12683_m.12683	DNA2_ACAPL	Plancitoxin-1 OS=Acanthaster planci OX=133434 PE=1 SV=1	1.8E-74	10.12
Nucleotide pyrophosphatase/phosphodiesterase family	Gene.26217_r_13397_g.26217_m.26217	PDE_MACLB	Venom phosphodiesterase OS=Macrovipera lebetina OX=8709 PE=1 SV=1	7.6E-36	4.58
Phospholipase A2 family. Group I subfamily. D49 sub-subfamily	Gene.30711_r_18012_g.30711_m.30711	PA2A_BUNMU	Acidic phospholipase A2 OS=Bungarus multicinctus OX=8616 PE=1 SV=2	1.1E-24	7.95
Phospholipase A2 family. Group III subfamily	Gene.30102_r_17363_g.30102_m.30102	PA23_HELSU	Phospholipase A2 isozymes PA3A/PA3B/PA5 OS=Heloderma	7.5E-30	144.13

			suspectum OX=8554 PE=1 SV=3		
Phospholipase B-like family	Gene.12079_r_3800_g.12079_m.12079	PLB_DRYCN	Phospholipase-B 81 OS=Drysdalia coronoides OX=66186 PE=1 SV=1	0	11.45
Venom metalloproteinase (M12B) family. P-III subfamily. P-iiia sub-subfamily	Gene.13186_r_4338_g.13186_m.13186	VM3K_NAJKA	Hemorrhagic metalloproteinase-disintegrin-like kaouthiagin OS=Naja kaouthia OX=8649 PE=1 SV=1	1.4E-29	83.90
Proteinase inhibitors					
Venom Kunitz-type family	Gene.32798_r_20368_g.32798_m.32798	VKT2_BITGA	Kunitz-type serine protease inhibitor bitisilin-2 OS=Bitis gabonica OX=8694 PE=1 SV=1	5.9E-20	38.81
Ion channel inhibitors					
CRISP family	Gene.16102_r_5875_g.16102_m.16102	CRVP_LATSE	Cysteine-rich venom protein latisemin OS=Laticauda semifasciata OX=8631 PE=2 SV=1	1.3E-26	12.67
	Gene.11853_r_3703_g.11853_m.11853	CRVP_HOPST	Cysteine-rich venom protein pseudechetoxin-like OS=Hoplocephalus stephensii OX=196418 PE=2 SV=1	3.6E-29	9.52
	Gene.9077_r_2517_g.9077_m.9077	CRVP_LEIMD	Cysteine-rich venom protein LEI1 (Fragment) OS=Leioheterodon madagascariensis OX=46577 PE=1 SV=1	2.5E-30	9.87
	Gene.21131_r_9101_g.21131_m.21131	CRVP_TRILK	CRISP/Allergen/PR-1 OS=Trittame loki OX=1295018 PE=1 SV=1	1.4E-33	464.73
	Gene.22696_r_10292_g.22696_m.22696	CRVP2_NAJKA	Cysteine-rich venom protein kaouthin-2 OS=Naja kaouthia OX=8649 PE=1 SV=2	2.7E-40	10.31
	Gene.26019_r_13221_g.26019_m.26019	CRVP_TELDH	Cysteine-rich venom protein TEL1 (Fragment) OS=Telescopus dhara OX=338837 PE=2 SV=1	1.6E-28	6.49
CRISP family. Venom allergen 5-like subfamily	Gene.44110_r_37299_g.44110_m.44110	VA543_SCODE	Scoloptoxin SSD43 OS=Scolopendra dehaani OX=2609776 PE=1 SV=1	2.4E-12	20.57
	Gene.33252_r_20934_g.33252_m.33252	VA5_VESVI	Venom allergen 5 OS=Vespula vidua OX=30215 PE=1 SV=1	2.4E-27	6.97

Scoloptoxin-05 family	Gene.14011_r_4742_g.14011_m.14011	TX51A_SCOMO	U-scoloptoxin(05)-Sm1a OS=Scolopendra morsitans OX=943129 PE=2 SV=1	8.6E-07	6.63
Scoloptoxin-11 family	Gene.38036_r_27351_g.38036_m.38036	TX14B_SCODE	Scoloptoxin SSD20 (Fragment) OS=Scolopendra dehaani OX=2609776 PE=1 SV=1	2.4E-27	7.46
Venom Kunitz-type family. Sea anemone type 2 potassium channel toxin subfamily	Gene.48512_r_45419_g.48512_m.48512	VKT1A_ANEVI	U-actitoxin-Avd3f OS=Anemonia viridis OX=51769 PE=3 SV=15,	1E-09	57.06
	Gene.37015_r_25865_g.37015_m.37015	VKTB_ANEVI	U-actitoxin-Avd3n OS=Anemonia viridis OX=51769 PE=3 SV=1	3.5E-14	20.64
	Gene.35176_r_23397_g.35176_m.35176	VKT5_ANEVI	U-actitoxin-Avd3h OS=Anemonia viridis OX=51769 PE=3 SV=14,	9E-20	7.23
Other toxins candidates					
AVIT (prokineticin) family	Gene.32199_r_19665_g.32199_m.32199	BO8_BOMOR	Prokineticin Bo8 OS=Bombina orientalis OX=8346 PE=1 SV=1	6.5E-07	76.69
EGF-like, Sushi, TSP type-1, LDL-receptor class A	Gene.17457_r_6644_g.17457_m.17457	CTX_SEPES	SE-cephalotoxin OS=Sepia esculenta OX=31210 PE=1 SV=1	1.1E-07	16.51
Elevenin - like	Gene.38214_r_27621_g.38214_m.38214	CELE_CONVC	Elevenin-Vc1 OS=Conus victoriae OX=319920 PE=1 SV=1	1.3E-06	11.90
Glycoprotein hormones subunit alpha family	Gene.21463_r_9336_g.21463_m.21463	CTHA2_CONVC	Thyrostimulin alpha-2 subunit OS=Conus victoriae OX=319920 PE=1 SV=1	4.9E-31	9.14
Glycoprotein hormones subunit beta family	Gene.35802_r_24219_g.35802_m.35802	CTHB5_CONVC	Thyrostimulin beta-5 subunit OS=Conus victoriae OX=319920 PE=2 SV=1	3E-17	3.73
IGFBP N-terminal, Kazal-like, Ig-like C2-type	Gene.20580_r_8675_g.20580_m.20580	IGFBP_CUPSA	Insulin-like growth factor-binding protein-related protein 1 OS=Cupiennius salei OX=6928 PE=2 SV=1	4.4E-68	42.79
TCTP family	Gene.14168_r_4820_g.14168_m.14168	TCTP_BRABE	Translationally-controlled tumor protein homolog OS=Branchiostoma belcheri OX=7741 PE=2 SV=1	2.2E-19	7.96
	Gene.16043_r_5846_g.16043_m.16043	TCTP_BRAOL	Translationally-controlled tumor protein homolog OS=Brassica oleracea OX=3712 GN=TCTP PE=2 SV=1	7.1E-23	325.64

Table S2. Toxin transcripts of *Cephalothrix hongkongiensis.* Unique toxins families are shown in bold.

**Protein family**	**Transcript ID**	**Uniprot accession**	**Description**	**E-value**	**Expression, TPM**
			Neurotoxins		
Neurotoxin 20 family	Gene.34245_c_hong_26879_g.34245_m.34245	TXCA_CAEEX	U3-aranetoxin-Ce1a OS=Caerostris extrusa OX=172846 PE=2 SV=1	4E-06	199.67
			Pore-forming toxins		
Actinoporin family. Conoidea subfamily	Gene.11883_c_hong_4214_g.11883_m.11883	ACTP1_TERAN	Tereporin-Ca1 (Fragment) OS=Terebra anilis OX=553697 PE=2 SV=1	1.8E-25	16.88
Actinoporin family. Sea anemone subfamily	Gene.26045_c_hong_15774_g.26045_m.26045	ACTP2_PHYSE	DELTA-alicitoxin-Pse1b OS=Phyllodiscus semoni OX=163701 PE=2 SV=1	4.9E-13	36.99
	Gene.35099_c_hong_28188_g.35099_m.35099	ACTP1_SAGRO	DELTA-sagatoxin-Srs1a OS=Sagartia rosea OX=396345 PE=1 SV=1	8.5E-19	1.96
MACPF domain	Gene.18196_c_hong_8305_g.18196_m.18196	TX60A_ACTVL	DELTA-thalatoxin-Avl2a OS=Actineria villosa OX=227975 PE=1 SV=1	9E-59	16.88
	Gene.5492_c_hong_1368_g.5492_m.5492	TX60B_PHYSE	DELTA-alicitoxin-Pse2b OS=Phyllodiscus semoni OX=163701 PE=1 SV=21,2	1E-65	14.88
	Gene.6822_c_hong_1836_g.6822_m.6822	TX60A_PHYSE	DELTA-alicitoxin-Pse2a OS=Phyllodiscus semoni OX=163701 PE=1 SV=1	7.5E-77	113.04
			Enzymes		
5'- nucleotidase family	Gene.8680_c_hong_2612_g.8680_m.8680	V5NTD_NAJAT	Snake venom 5'-nucleotidase (Fragment) OS=Naja atra OX=8656 PE=1 SV=1	8E-153	5.61
Arthropod phospholipase D family. Class II subfamily	Gene.31998_c_hong_23588_g.31998_m.31998	A1LB2_LOXAR	Dermonecrotic toxin larsictox-alphaib2bii (Fragment) OS=Loxosceles arizonica OX=196454 PE=2 SV=1	6.9E-26	5.62

	Gene.18047_c_hong_8189_g.18047_m.18047	A1I1_LOXSP	Dermonecrotic toxin lspasictox-alphaia2i (Fragment) OS=Loxosceles spadicea OX=571530 PE=2 SV=1	3.9E-47	14.08
Dnase II family	Gene.10361_c_hong_3406_g.10361_m.10361	DNA2_ACAPL	Plancitoxin-1 OS=Acanthaster planci OX=133434 PE=1 SV=1	2.4E-71	3.65
Flavin monoamine oxidase family. FIG1 subfamily	Gene.29350_c_hong_19924_g.29350_m.29350	OXLA_CROAT	L-amino-acid oxidase apoxin-1 OS=Crotalus atrox OX=8730 PE=1 SV=2	2.9E-20	3.10
Glycosyl hydrolase 56 family	Gene.17971_c_hong_8131_g.17971_m.17971	HYAL1_MESMA	Hyaluronidase-1 OS=Mesobuthus martensii OX=34649 PE=1 SV=2	1.1E-54	5.89
Nucleotide pyrophosphatase/phosphodiesterase family	Gene.11880_c_hong_4213_g.11880_m.11880	PDE_CRODO	Venom phosphodiesterase cdcpde OS=Crotalus durissus collilineatus OX=221569 PE=1 SV=1	2.1E-66	3.74
	Gene.27311_c_hong_17262_g.27311_m.27311	PDE1_CROAD	Venom phosphodiesterase 1 OS=Crotalus adamanteus OX=8729 PE=1 SV=2	1.7E-66	3.62
Phospholipase A2 family	Gene.24117_c_hong_13701_g.24117_m.24117	PA21_SCODE	Phospholipase A2 SSD387 OS=Scolopendra dehaani OX=2609776 PE=1 SV=1	1.8E-25	5.78
Phospholipase A2 family. Group III subfamily	Gene.17905_c_hong_8085_g.17905_m.17905	PA23_HELSU	Phospholipase A2 isozymes PA3A/PA3B/PA5 OS=Heloderma suspectum OX=8554 PE=1 SV=3	1.4E-20	39.35
	Gene.16417_c_hong_7078_g.16417_m.16417	PA2A2_HELSU	Acidic phospholipase A2 PA4 OS=Heloderma suspectum OX=8554 PE=1 SV=2	3.2E-36	8.47

Venom metalloproteinase (M12B) family. P-III subfamily. P-iiia sub-subfamily	Gene.47013_c_hong_50313_g.47013_m.47013	VM3_CERRY	Zinc metalloproteinase-disintegrin-like OS=Cerberus rynchops OX=46267 PE=1 SV=1	6.6E-14	6.06
**Venom metalloproteinase (M12B) family. P-II subfamily. P-iid sub-subfamily**	**Gene.32433_c_hong_24210_g.32433_m.32433**	**VM2SA_GLOSA**	**Zinc metalloproteinase/disintegrin OS=Gloydius saxatilis OX=92067 PE=2 SV=1**	**1.4E-11**	**3.58**
			Ion channel inhibitor activity		
CRISP family	Gene.21111_c_hong_10759_g.21111_m.21111	CRVP_OXYMI	Cysteine-rich venom protein pseudechetoxin-like OS=Oxyuranus microlepidotus OX=111177 PE=2 SV=1	6.4E-30	4.19
	Gene.749_c_hong_89_g.749_m.749	CRVP_HOPST	Cysteine-rich venom protein pseudechetoxin-like OS=Hoplocephalus stephensii OX=196418 PE=2 SV=1	2.7E-30	27.92
	Gene.22720_c_hong_12307_g.22720_m.22720	CRVP_AUSSU	Cysteine-rich venom protein OS=Austrelaps superbus OX=29156 PE=2 SV=1	9.2E-36	3.07
	Gene.6717_c_hong_1800_g.6717_m.6717	CRVP_ERYPO	Cysteine-rich venom protein LIO1 (Fragment) OS=Erythrolamprus poecilogyrus OX=338838 PE=2 SV=1	7.4E-37	4.53
	Gene.12301_c_hong_4442_g.12301_m.12301	MR30_CONMR	Cysteine-rich venom protein Mr30 OS=Conus marmoreus OX=42752 PE=1 SV=1	2.1E-39	5.22
CRISP family. Venom allergen 5-like subfamily	Gene.38009_c_hong_33066_g.38009_m.38009	VA51_VESCR	Venom allergen 5.01 OS=Vespa crabro OX=7445 PE=1 SV=1	7.5E-15	1.45

	Gene.15844_c_hong_6675_g.15844_m.15844	VA576_SCODE	Scoloptoxin SSD976 OS=Scolopendra dehaani OX=2609776 PE=1 SV=1	3.2E-25	6.48
Scoloptoxin-05 family	Gene.29823_c_hong_20580_g.29823_m.29823	TX51A_ETHRU	U-scoloptoxin(05)-Er1a OS=Ethmostigmus rubripes OX=62613 PE=2 SV=1	1E-06	2.87
Sea anemone type 5 potassium channel toxin family	Gene.30277_c_hong_21219_g.30277_m.30277	KV53_BUNCI	Kappa-actitoxin-Bcs4a OS=Bunodosoma caissarum OX=31165 PE=1 SV=1	2.6E-19	9.35
			Hemostasis impairing toxin		
Snaclec family	Gene.43733_c_hong_43800_g.43733_m.43733	SLUA_DEIAC	Snaclec agkisacutacin subunit A OS=Deinagkistrodon acutus OX=36307 PE=1 SV=2	1.7E-09	4.39
	Gene.48482_c_hong_53305_g.48482_m.48482	VA5_VESPE	Venom allergen 5 OS=Vespula pensylvanica OX=30213 PE=1 SV=1	5E-10	3.00
	Gene.7929_c_hong_2279_g.7929_m.7929	SLLC2_DABSI	Snaclec coagulation factor X-activating enzyme light chain 2 OS=Daboia siamensis OX=343250 GN=LC2 PE=1 SV=1	8.1E-09	14.65
SNTX/VTX toxin family	Gene.32124_c_hong_23751_g.32124_m.32124	CTXB_SCOPL	Cytolytic toxin-beta OS=Scorpaena plumieri OX=274700 PE=1 SV=1	4.5E-19	1.96
	Gene.938_c_hong_118_g.938_m.938	CTXA_SCOPL	Cytolytic toxin-alpha OS=Scorpaena plumieri OX=274700 PE=1 SV=1	4.1E-52	27.73
			Other toxins candidates		
Conotoxin-like	Gene.35778_c_hong_29319_g.35778_m.35778	CXE3_CONIM	Conotoxin Im14.3 OS=Conus imperialis OX=35631 PE=1 SV=1	1E-07	2.98
EGF-like, Sushi, TSP type-1,	Gene.12659_c_hong_4652_	CTX_SEPES	SE-cephalotoxin OS=Sepia esculenta	4.7E-73	8.01

LDL-receptor class A	g.12659_m.12659		OX=31210 PE=1 SV=1		
Elevenin-like	Gene.46986_c_hong_50253_g.46986_m.46986	CELE_CONVC	Elevenin-Vc1 OS=Conus victoriae OX=319920 PE=1 SV=1	2E-07	5.19
IGFBP N-terminal domain, Kazal-like, Ig-like C2-type	Gene.20111_c_hong_9872_g.20111_m.20111	IGFBP_CUPSA	Insulin-like growth factor-binding protein-related protein 1 OS=Cupiennius salei OX=6928 PE=2 SV=1	4.6E-62	14.00
Vasopressin/oxytocin family	Gene.40543_c_hong_37563_g.40543_m.40543	CESS2_CONMO	Conopressin/conophysin, isoform 2 (Fragment) OS=Conus monile OX=351660 PE=2 SV=1	2.6E-24	3.22

Table S3. Toxin transcripts of *Cephalothrix* cf. *simula*. Unique toxins families are shown in bold. The expression might be quantified using Illumina reads only. No expression values were obtained for toxins transcripts from MinIon Oxford nanopore sequencing; they were assigned as NA.

Protein family	Transcript ID	Uniprot accession	Description	E-value	Expression, TPM
Neurotoxins					
Neurotoxin 20 family	Gene.485063_csim_minion_347459_g.485063_m.485063	TXCA_CAEEX	U3-aranetoxin-Ce1a OS=Caerostris extrusa OX=172846 PE=2 SV=1	4E-06	30.77
Pore-forming toxins					
Actinoporin family. Conoidea subfamily	Gene.35246_csim_minion_9736_g.35246_m.35246	ACTP1_TERSU	Tereporin-Ts1 (Fragment) OS=Terebra subulata OX=89435 PE=2 SV=1	2.9E-13	NA
	Gene.335613_csim_minion_180378_g.335613_m.335613	ACTP1_TERAN	Tereporin-Ca1 (Fragment) OS=Terebra anilis OX=553697 PE=2 SV=1	2.9E-26	NA
Actinoporin family. Sea anemone subfamily	Gene.474824_csim_minion_331394_g.474824_m.474824	ACTP5_ACTEQ	DELTA-actitoxin-Aeq1b OS=Actinia equina OX=6106 PE=2 SV=1	1.4E-09	NA
	Gene.407909_csim_minion_249004_g	ACTP2_PHYSE	DELTA-alicitoxin-Pse1b OS=Phyllodiscus semoni	2E-15	NA

	.407909_m.407909		OX=163701 PE=2 SV=1		
MACPF domain	Gene.301043_csim_minion_153953_g.301043_m.301043	TX60A_ACTVL	DELTA-thalatoxin-Avl2a OS=Actineria villosa OX=227975 PE=1 SV=1	4.6E-66	31.85
	Gene.273396_csim_minion_135569_g.273396_m.273396	TX60A_PHYSE	DELTA-alicitoxin-Pse2a OS=Phyllodiscus semoni OX=163701 PE=1 SV=1	4.3E-77	115.63
	Gene.293181_csim_minion_148573_g.293181_m.293181	TX60B_PHYSE	DELTA-alicitoxin-Pse2b OS=Phyllodiscus semoni OX=163701 PE=1 SV=2	1.2E-80	0.58
Enzymes					
5'- nucleotidase family	Gene.197718_csim_minion_86712_g.197718_m.197718	V5NTD_GLOBR	Snake venom 5'-nucleotidase OS=Gloydius brevicaudus OX=259325 PE=2 SV=1	3.2E-22	NA
Arthropod phospholipase D family. Class II subfamily	Gene.464893_csim_minion_317229_g.464893_m.464893	A21_LOXVA	Dermonecrotic toxin lvsictox-alphaii1 (Fragment) OS=Loxosceles variegata OX=571533 PE=2 SV=1	7.1E-11	NA
	Gene.510941_csim_minion_397287_g.510941_m.510941	B1R_LOXAS	Dermonecrotic toxin lafsictox-betaie1 (Fragment) OS=Loxosceles aff. Spinulosa (strain GJB-2008) OX=575951 PE=2 SV=1	8.6E-12	NA
	Gene.403668_csim_minion_244462_g.403668_m.403668	A1O_LOXGA	Dermonecrotic toxin lgsictox-alphaic1 OS=Loxosceles gaucho OX=58216 PE=1 SV=1	1.5E-14	NA
	Gene.381627_csim_minion_221329_g.381627_m.381627	B1H2_LOXIN	Dermonecrotic toxin lisictox-betaia1ii OS=Loxosceles intermedia OX=58218 PE=2 SV=1	4.7E-15	NA

	Gene.372490 _csim_minion_212540_g .372490_m.37 2490	A1OB3_LOXVA	Dermonecrotic toxin lvsictox-alphaic1biii (Fragment) OS=Loxosceles variegata OX=571533 PE=2 SV=1	4.2E-20	NA
	Gene.423589 _csim_minion_266101_g .423589_m.42 3589	A1IA3_LOXIN	Dermonecrotic toxin lisictox-alphaia2aiii (Fragment) OS=Loxosceles intermedia OX=58218 PE=2 SV=1	8.6E-21	NA
	Gene.380591 _csim_minion_220301_g .380591_m.38 0591	A411_LOXHI	Dermonecrotic toxin lhsictox-alphaiv1i (Fragment) OS=Loxosceles hirsuta OX=571525 PE=2 SV=1	1.6E-21	NA
	Gene.503547 _csim_minion_382170_g .503547_m.50 3547	B1H_LOXLA	Dermonecrotic toxin llsictox-betaia1 OS=Loxosceles laeta OX=58217 PE=1 SV=1	1.7E-20	NA
	Gene.290660 _csim_minion_146874_g .290660_m.29 0660	A1I1_LOXSP	Dermonecrotic toxin lspasictox-alphaia2i (Fragment) OS=Loxosceles spadicea OX=571530 PE=2 SV=1	4E-47	NA
	Gene.387485 _csim_minion_227187_g .387485_m.38 7485	A1IB1_LOXIN	Dermonecrotic toxin lisictox-alphaia2bi OS=Loxosceles intermedia OX=58218 PE=2 SV=1	5.4E-54	NA
Dnase II family	Gene.354317 _csim_minion_196206_g .354317_m.35 4317	DNA2_ACAPL	Plancitoxin-1 OS=Acanthaster planci OX=133434 PE=1 SV=1	1.3E-71	2.96
Flavin monoamine oxidase family. FIG1 subfamily	Gene.138452 _csim_minion_54012_g. 138452_m.13 8452	OXLA_MICMP	L-amino acid oxidase OS=Micrurus mipartitus OX=430902 PE=1 SV=1	2.3E-14	NA
Glycosyl hydrolase 56 family	Gene.408049 _csim_minion_249149_g	HUGA_APIME	Hyaluronidase OS=Apis mellifera OX=7460 PE=1 SV=1	3.1E-18	NA

	.408049_m.408049				
	Gene.399637_csim_minion_239919_g.399637_m.399637	HYAL_LOXIN	Hyaluronidase OS=Loxosceles intermedia OX=58218 PE=2 SV=1	7E-23	NA
	Gene.342583_csim_minion_186216_g.342583_m.342583	HYAL1_MESMA	Hyaluronidase-1 OS=Mesobuthus martensii OX=34649 PE=1 SV=2	4.7E-51	NA
Nucleotide pyrophosphatase/phosphodiesterase family	Gene.10925_csim_minion_2435_g.10925_m.10925	PDE_MACLB	Venom phosphodiesterase OS=Macrovipera lebetina OX=8709 PE=1 SV=1	3.2E-60	0.45
**Peptidase S1 family. Snake venom subfamily**	**Gene.450589_csim_minion_298071_g.450589_m.450589**	**VSPPA_GLOBR**	**Venom plasminogen activator Haly-PA OS=Gloydius brevicaudus OX=259325 PE=1 SV=2**	**2.5E-16**	**NA**
Phospholipase A2 family	Gene.437846_csim_minion_282313_g.437846_m.437846	PA2_CONGI	Phospholipase A2 A2-actitoxin-Cgg2a OS=Condylactis gigantea OX=47073 PE=1 SV=1	2.5E-13	NA
	Gene.441169_csim_minion_286288_g.441169_m.441169	PA21_SCODE	Phospholipase A2 SSD387 OS=Scolopendra dehaani OX=2609776 PE=1 SV=1	3.9E-28	35.91
Phospholipase A2 family. Group I subfamily. D49 sub-subfamily	Gene.524403_csim_minion_430111_g.524403_m.52440	PA2A2_PSETE	Acidic phospholipase A2 2 OS=Pseudonaja textilis OX=8673 PE=1 SV=1	8E-07	NA
	Gene.518466_csim_minion_414418_g.518466_m.518466	PA2B2_BUNCE	Basic phospholipase A2 beta-bungarotoxin A2 chain OS=Bungarus caeruleus OX=132961 PE=2 SV=1	3E-08	NA
	Gene.466258_csim_minion_319116_g.466258_m.466258	PA2A1_PSETE	Acidic phospholipase A2 1 OS=Pseudonaja textilis OX=8673 PE=1 SV=1	2.9E-09	NA

Phospholipase A2 family. Group III subfamily	Gene.436441_csim_minion_280594_g.436441_m.436441	PA23_HELSU	Phospholipase A2 isozymes PA3A/PA3B/PA5 OS=Heloderma suspectum OX=8554 PE=1 SV=3	8.4E-30	2.50
Phospholipase B-like family	Gene.90104_csim_minion_31095_g.90104_m.90104	PLB_DRYCN	Phospholipase-B 81 OS=Drysdalia coronoides OX=66186 PE=1 SV=1	5.3E-170	42.77
Venom metalloproteinase (M12B) family. P-III subfamily. P-iiia sub-subfamily	Gene.518063_csim_minion_413463_g.518063_m.518063	VM3A_NAJAT	Zinc metalloproteinase-disintegrin-like atrase-A OS=Naja atra OX=8656 PE=2 SV=1	9.5E-38	1.11
Proteinase inhibitors					
Natriuretic peptide family	Gene.299081_csim_minion_152562_g.299081_m.299081	SVMI1_CERCE	Snake venom metalloprotease inhibitor 02A10 OS=Cerastes cerastes OX=8697 GN=Svmpi-Cce12 PE=1 SV=1	5E-07	226.81
Venom Kunitz-type family	Gene.217819_csim_minion_99052_g.217819_m.217819	VKT_ERIMA	Kunitz-type serine protease inhibitor OS=Eristicophis macmahoni OX=110227 PE=1 SV=1	5E-06	NA
	Gene.217316_csim_minion_98765_g.217316_m.217316	VKTCI_OPHHA	Kunitz-type serine protease inhibitor OS=Ophiophagus hannah OX=8665 PE=1 SV=2	2.4E-09	2.95
	Gene.4163_csim_minion_791_g.4163_m.4163	VKT2_BITGA	Kunitz-type serine protease inhibitor bitisilin-2 OS=Bitis gabonica OX=8694 PE=1 SV=1	3.8E-19	1.14
**Venom Kunitz-type family. 03 (sub-Kunitz) subfamily**	**Gene.520922_csim_minion_420428_g.520922_m.520922**	**VKT76_CYRHA**	**Kunitz-type serine protease inhibitor HNTX-0109076 (Fragment) OS=Cyriopagopus hainanus OX=209901 PE=2 SV=1**	**1.2E-12**	**0.15**

Venom Kunitz-type family. Sea anemone type 2 potassium channel toxin subfamily	Gene.133532_csim_minion_51501_g.133532_m.133532	VKT1_ANTAF	PI-actitoxin-Axm2a OS=Anthopleura aff. Xanthogrammica OX=152178 PE=1 SV=1	2E-07	NA
			Ion channel inhibitor activity		
CRISP family	Gene.212790_csim_minion_95926_g.212790_m.212790	CRVP_LEIMD	Cysteine-rich venom protein LEI1 (Fragment) OS=Leioheterodon madagascariensis OX=46577 PE=1 SV=1	5.3E-09	NA
	Gene.430137_csim_minion_273348_g.430137_m.430137	VA3_DINQU	Venom allergen 3 homolog OS=Dinoponera quadriceps OX=609295 PE=2 SV=1	1E-08	NA
	Gene.405636_csim_minion_246603_g.405636_m.405636	CRVP_DEMVE	Cysteine-rich venom protein OS=Demansia vestigiata OX=412038 PE=2 SV=1	1.5E-12	NA
	Gene.260819_csim_minion_127234_g.260819_m.260819	CRVP_PSETE	Cysteine-rich venom protein pseudechetoxin-like OS=Pseudonaja textilis OX=8673 PE=1 SV=1	1.7E-12	NA
	Gene.435671_csim_minion_279641_g.435671_m.435671	CRVP_TRILK	CRISP/Allergen/PR-1 OS=Trittame loki OX=1295018 PE=1 SV=1	8.4E-15	NA
	Gene.15278_csim_minion_3622_g.15278_m.15278	CRVP_DRYCN	Cysteine-rich venom protein OS=Drysdalia coronoides OX=66186 PE=1 SV=1	5E-19	NA
	Gene.527762_csim_minion_440812_g.527762_m.527762	CRVP_PSEAU	Cysteine-rich venom protein pseudechetoxin OS=Pseudechis australis OX=8670 PE=1 SV=1	3.1E-24	NA
	Gene.298733_csim_minion_152323_g.298733_m.298733	VA576_SCODE	Scoloptoxin SSD976 OS=Scolopendra dehaani OX=2609776 PE=1 SV=1	4.5E-29	73.35

	Gene.164601_csim_minion_67500_g.164601_m.164601	CRVP2_NAJKA	Cysteine-rich venom protein kaouthin-2 OS=Naja kaouthia OX=8649 PE=1 SV=2	3.4E-31	5.50
	Gene.178202_csim_minion_75128_g.178202_m.178202	MR30_CONMR	Cysteine-rich venom protein Mr30 OS=Conus marmoreus OX=42752 PE=1 SV=1	4.4E-28	2.02
	Gene.10434_csim_minion_2310_g.10434_m.10434	CRVP_HOPST	Cysteine-rich venom protein pseudechetoxin-like OS=Hoplocephalus stephensii OX=196418 PE=2 SV=1	4.3E-30	16.04
	Gene.353457_csim_minion_195465_g.353457_m.353457	TX31_CONTE	Cysteine-rich venom protein OS=Conus textile OX=6494 PE=1 SV=1	2.7E-31	1.36
	Gene.305948_csim_minion_157402_g.305948_m.305948	CRVP_CERRY	Cysteine-rich venom protein OS=Cerberus rynchops OX=46267 PE=1 SV=1	1.8E-34	1.57
	Gene.321837_csim_minion_169492_g.321837_m.321837	CRVP_ERYPO	Cysteine-rich venom protein LIO1 (Fragment) OS=Erythrolamprus poecilogyrus OX=338838 PE=2 SV=1	1.2E-36	21.73
CRISP family. Venom allergen 5-like subfamily	Gene.418340_csim_minion_260429_g.418340_m.418340	VA5_LYCSI	Venom allergen 5 OS=Lycosa singoriensis OX=434756 PE=2 SV=1	4E-11	NA
	Gene.363965_csim_minion_204653_g.363965_m.363965	VA5_POLFU	Venom allergen 5 OS=Polistes fuscatus OX=30207 PE=1 SV=1	6.1E-09	NA
	Gene.147066_csim_minion_58363_g.	VA53_DOLMA	Venom allergen 5.02 (Fragment) OS=Dolichovespula	6.3E-15	0.15

	147066_m.147066		maculata OX=7441 PE=1 SV=3		
Jellyfish toxin family. Type II subfamily	Gene.464452_csim_minion_316670_g.464452_m.464452	JTX1_CARRA	Toxin crtx-A OS=Carybdea rastonii OX=78582 PE=1 SV=1	1.7E-21	NA
	Gene.216327_csim_minion_98149_g.216327_m.216327	JTX1_CARRA	Toxin crtx-A OS=Carybdea rastonii OX=78582 PE=1 SV=1	2.7E-12	NA
Scoloptoxin-05 family	Gene.197801_csim_minion_86749_g.197801_m.197801	TX51A_ETHRU	U-scoloptoxin(05)-Er1a OS=Ethmostigmus rubripes OX=62613 PE=2 SV=1	6E-08	3.99
Scoloptoxin-11 family	Gene.397028_csim_minion_237164_g.397028_m.397028	TXB3A_SCODE	U-scoloptoxin(11)-Ssd3a OS=Scolopendra dehaani OX=2609776 PE=1 SV=1	3E-07	0.09
Sea anemone type 5 potassium channel toxin family	Gene.517497_csim_minion_412076_g.517497_m.517497	KV51_METSE	U-metritoxin-Msn2a OS=Metridium senile OX=6116 PE=3 SV=1	1.1E-15	66.35
	Gene.496728_csim_minion_368326_g.496728_m.496728	KV53_BUNCI	Kappa-actitoxin-Bcs4a OS=Bunodosoma caissarum OX=31165 PE=1 SV=1	2.4E-19	1.41
Venom Kunitz-type family. Sea anemone type 2 potassium channel toxin subfamily	Gene.323953_csim_minion_171126_g.323953_m.323953	VKTG_ANEVI	U-actitoxin-Avd3s (Fragment) OS=Anemonia viridis OX=51769 PE=2 SV=1	2.8E-10	NA
			Hemostasis impairing toxin		
**Ficolin lectin family. Veficolin subfamily**	**Gene.445543_csim_minion_291627_g.445543_m.445543**	**FCNV3_CERRY**	**Ryncolin-3 OS=Cerberus rynchops OX=46267 PE=1 SV=1**	**8.4E-33**	**1.11**
Snaclec family	Gene.355855_csim_minion_197543_g.355855_m.355855	SLAB3_TRIST	Snaclec stejaggregin-A subunit beta-3 OS=Trimeresurus stejnegeri OX=39682 PE=2 SV=1	2.9E-12	NA

	Gene.14062_csim_minion_3288_g.14062_m.14062	SLEB_CALRH	Snaclec rhodocetin subunit beta OS=Calloselasma rhodostoma OX=8717 PE=1 SV=1	3E-06	0.11
	Gene.424617_csim_minion_267267_g.424617_m.424617	SLB2_MACLB	Snaclec B2 OS=Macrovipera lebetina OX=8709 PE=2 SV=1	4E-07	1.25
	Gene.96560_csim_minion_33927_g.96560_m.96560	SLLC2_MACLB	Snaclec coagulation factor X-activating enzyme light chain 2 OS=Macrovipera lebetina OX=8709 GN=LC2 PE=1 SV=1	3E-08	4.44
	Gene.67427_csim_minion_21579_g.67427_m.67427	SLLC2_DABSI	Snaclec coagulation factor X-activating enzyme light chain 2 OS=Daboia siamensis OX=343250 GN=LC2 PE=1 SV=1	8.6E-09	NA
SNTX/VTX toxin family	Gene.45640_csim_minion_13412_g.45640_m.45640	STXA_SYNVE	Neoverrucotoxin subunit alpha OS=Synanceia verrucosa OX=51996 PE=1 SV=1	7.2E-15	NA
	Gene.19568_csim_minion_4884_g.19568_m.19568	CTXB_SCOPL	Cytolytic toxin-beta OS=Scorpaena plumieri OX=274700 PE=1 SV=1	1.4E-19	0.09
	Gene.429229_csim_minion_272364_g.429229_m.429229	STXB_SYNVE	Neoverrucotoxin subunit beta OS=Synanceia verrucosa OX=51996 PE=1 SV=1	2.8E-27	1.56
	Gene.17739_csim_minion_4362_g.17739_m.17739	CTXA_SCOPL	Cytolytic toxin-alpha OS=Scorpaena plumieri OX=274700 PE=1 SV=1	3.7E-52	64.77
True venom lectin family	Gene.200635_csim_minion_88492_g.200635_m.200635	LECG1_BUNFA	C-type lectin bfl-1 OS=Bungarus fasciatus OX=8613 PE=2 SV=1	6E-07	NA

	Gene.381021_csim_minion_220751_g.381021_m.381021	LECM2_BUNMU	C-type lectin BML-2 OS=Bungarus multicinctus OX=8616 PE=1 SV=1	5E-08	NA
	Gene.347148_csim_minion_190074_g.347148_m.347148	LECM1_HYDHA	C-type lectin 1 OS=Hydrophis hardwickii OX=8781 PE=2 SV=1	2E-08	5.66
			Other toxins candidates		
AVIT (prokineticin) family	Gene.306077_csim_minion_157513_g.306077_m.306077	MIT1_DENPO	Toxin MIT1 OS=Dendroaspis polylepis polylepis OX=8620 PE=1 SV=3	1E-08	NA
EGF-like, Sushi, TSP type-1, LDL-receptor class A	Gene.154083_csim_minion_61961_g.154083_m.154083	CTX_SEPES	SE-cephalotoxin OS=Sepia esculenta OX=31210 PE=1 SV=1	1.6E-35	1.30
Elevenin-like	Gene.291057_csim_minion_147124_g.291057_m.291057	CELE_CONVC	Elevenin-Vc1 OS=Conus victoriae OX=319920 PE=1 SV=1	3E-07	NA
IGFBP N-terminal, Kazal-like, Ig-like C2-type	Gene.254428_csim_minion_123013_g.254428_m.254428	IGFBP_CUPSA	Insulin-like growth factor-binding protein-related protein 1 OS=Cupiennius salei OX=6928 PE=2 SV=1	4.6E-62	15.78
Venom complement C3 homolog family	Gene.461311_csim_minion_312333_g.461311_m.461311	VCO32_AUSSU	A.superbus venom factor 2 OS=Austrelaps superbus OX=29156 PE=2 SV=1	2.8E-13	NA

Table S4. Toxin transcripts of *Tubulanus polymorphus*. Unique toxins families are shown in bold.

Protein family	Transcript ID	Uniprot accession	Description	E-value	Expression, TPM
			Pore-forming toxins		
Actinoporin family. Conoidea subfamily	Gene.32873_r_15596_g.32873_m.32873	ACTP1_TERAN	Tereporin-Ca1 (Fragment) OS=Terebra anilis OX=553697 PE=2 SV=1	5.4E-36	62.64
			Enzymes		

Arthropod phospholipase D family. Class II subfamily	Gene.48334_r_35867_g.48334_m.48334	B1T1_LOXSN	Dermonecrotic toxin lspisictox-betaie3i (Fragment) OS=Loxosceles spinulosa OX=571532 PE=2 SV=1	4.5E-12	13.49
	Gene.40754_r_23774_g.40754_m.40754	B1R2_LOXSN	Dermonecrotic toxin lspisictox-betaie1ii (Fragment) OS=Loxosceles spinulosa OX=571532 PE=2 SV=1	7.3E-15	9.10
	Gene.31713_r_14663_g.31713_m.31713	A412_LOXHI	Dermonecrotic toxin lhsictox-alphaiv1ii (Fragment) OS=Loxosceles hirsuta OX=571525 PE=2 SV=1	1.2E-41	6.59
	Gene.27650_r_11664_g.27650_m.27650	A1O_LOXGA	Dermonecrotic toxin lgsictox-alphaic1 OS=Loxosceles gaucho OX=58216 PE=1 SV=1	4.8E-48	4.84
Arthropod phospholipase D family. Class II subfamily. Class iia sub-subfamily	Gene.20564_r_7470_g.20564_m.20564	A51_LOXIN	Dermonecrotic toxin lisictox-alphav1 OS=Loxosceles intermedia OX=58218 PE=1 SV=1	3.6E-48	375.93
Dnase II family	Gene.21807_r_8136_g.21807_m.21807	DNA2_ACAPL	Plancitoxin-1 OS=Acanthaster planci OX=133434 PE=1 SV=1	3.2E-70	22.11
**GGT_peptidase domain**	**Gene.7717_r_1895_g.7717_m.7717**	**TX14A_SCODE**	**Scoloptoxin SSD14 OS=Scolopendra dehaani OX=2609776 PE=1 SV=1**	**2.2E-154**	**4.21**
Glutaminyl-peptide cyclotransferase family	Gene.45237_r_30379_g.45237_m.45237	QPCT_BOIDE	Glutaminyl-peptide cyclotransferase OS=Boiga dendrophila OX=46286 GN=QPCT PE=2 SV=1	4.4E-10	8.08
Natterin family	Gene.36879_r_19297_g.36879_m.36879	NATT3_THANI	Natterin-3 OS=Thalassophryne nattereri OX=289382 PE=1 SV=1	3E-12	4.58

	Gene.25133_r_10065_g.25133_m.25133	NATT4_THANI	Natterin-4 OS=Thalassophryne nattereri OX=289382 PE=2 SV=1	6E-16	6.23
Phospholipase A2 family	Gene.36857_r_19278_g.36857_m.36857	PA2_URTCR	Phospholipase A2 A2-actitoxin-Ucs2a OS=Urticina crassicornis OX=45621 PE=1 SV=1	7.1E-22	57.12
	Gene.11763_r_3435_g.11763_m.11763	PA2_SCOVI	Phospholipase A2 Scol/Pla OS=Scolopendra viridis OX=118503 PE=1 SV=1	1.2E-23	17.73
	Gene.34244_r_16760_g.34244_m.34244	PA21_SCODE	Phospholipase A2 SSD387 OS=Scolopendra dehaani OX=2609776 PE=1 SV=1	2.1E-29	9.31
Phospholipase A2 family. Group I subfamily. D49 sub-subfamily	Gene.20339_r_7345_g.20339_m.20339	PA2HB_OXYSC	Neutral phospholipase A2 homolog taipoxin beta chain 1 OS=Oxyuranus scutellatus scutellatus OX=8667 PE=1 SV=2	6.3E-19	40.41
	Gene.38345_r_20954_g.38345_m.38345	PA2A6_TROCA	Acidic phospholipase A2 6 OS=Tropidechis carinatus OX=100989 PE=2 SV=1	2E-21	35.95
	Gene.37234_r_19686_g.37234_m.37234	PA22_OXYSC	Phospholipase A2 OS2 OS=Oxyuranus scutellatus scutellatus OX=8667 PE=1 SV=2	4.3E-23	35.72
	Gene.26820_r_11136_g.26820_m.26820	PA2B_MICCO	Basic phospholipase A2 OS=Micrurus corallinus OX=54390 PE=1 SV=1	4.8E-25	27.75
Phospholipase A2 family. Group II subfamily. D49 sub-subfamily	Gene.33600_r_16227_g.33600_m.33600	PA2BA_CRODU	Phospholipase A2 crotoxin basic chain cba2 OS=Crotalus durissus terrificus OX=8732 PE=1 SV=1	8.3E-17	24.36

	Gene.40578_r_23581_g.40578_m.40578	PA2B2_DABRR	Basic phospholipase A2 Drk-b2 OS=Daboia russelii OX=8707 PE=1 SV=1	4E-21	16.13
Phospholipase A2 family. Group III subfamily	Gene.9410_r_2486_g.9410_m.9410	PA23_HELSU	Phospholipase A2 isozymes PA3A/PA3B/PA5 OS=Heloderma suspectum OX=8554 PE=1 SV=3	1.1E-19	23.19
Phospholipase B-like family	Gene.7245_r_1736_g.7245_m.7245	PLB_DRYCN	Phospholipase-B 81 OS=Drysdalia coronoides OX=66186 PE=1 SV=1	0	12.37
Venom metalloproteinase (M12B) family. P-III subfamily	Gene.8763_r_2255_g.8763_m.8763	VM38_DRYCN	Zinc metalloproteinase-disintegrin-like MTP8 OS=Drysdalia coronoides OX=66186 PE=1 SV=1	4.4E-70	15.46
Venom metalloproteinase (M12B) family. P-III subfamily. P-iiia sub-subfamily	Gene.15411_r_4963_g.15411_m.15411	VM3K_NAJKA	Hemorrhagic metalloproteinase-disintegrin-like kaouthiagin OS=Naja kaouthia OX=8649 PE=1 SV=1	1.8E-33	150.29
Proteinase inhibitors					
Venom Kunitz-type family	Gene.40270_r_23201_g.40270_m.40270	VKT3_PSETT	Kunitz-type serine protease inhibitor textilinin-3 OS=Pseudonaja textilis textilis OX=169397 PE=2 SV=1	1.2E-12	10.68
	Gene.46405_r_32385_g.46405_m.46405	VKT1_VIPAA	Kunitz-type serine protease inhibitor 1 OS=Vipera ammodytes ammodytes OX=8705 PE=1 SV=2	2.2E-18	41.66
Ion channel inhibitor activity					
CRISP family	Gene.53044_r_45762_g.53044_m.53044	CRVP_OPHHA	Cysteine-rich venom protein ophanin OS=Ophiophagus hannah OX=8665 PE=1 SV=1	5.9E-06	15.44

	Gene.33069_r_15764_g.33069_m.33069	VA3_SOLIN	Venom allergen 3 OS=Solenopsis invicta OX=13686 PE=1 SV=2	2.7E-31	32.60
	Gene.22507_r_8513_g.22507_m.22507	MR30_CONMR	Cysteine-rich venom protein Mr30 OS=Conus marmoreus OX=42752 PE=1 SV=1	1.3E-33	15.77
	Gene.25045_r_10009_g.25045_m.25045	CRVP_TRILK	CRISP/Allergen/PR-1 OS=Trittame loki OX=1295018 PE=1 SV=1	1.4E-35	19.85
CRISP family. Venom allergen 5-like subfamily	Gene.44509_r_29206_g.44509_m.44509	VA5_POLDO	Venom allergen 5 OS=Polistes dominula OX=743375 PE=1 SV=2	3.9E-08	20.85
	Gene.44041_r_28460_g.44041_m.44041	VA5_VESMG	Venom allergen 5 OS=Vespa magnifica OX=202807 PE=1 SV=2	1.4E-08	4.36
	Gene.21335_r_7884_g.21335_m.21335	VA576_SCODE	Scoloptoxin SSD976 OS=Scolopendra dehaani OX=2609776 PE=1 SV=1	1.9E-26	63.60
Venom Kunitz-type family. Sea anemone type 2 potassium channel toxin subfamily	Gene.37385_r_19844_g.37385_m.37385	VKT9_ANEVI	U-actitoxin-Avd3l OS=Anemonia viridis OX=51769 PE=3 SV=1	8E-15	151.04
	Gene.35073_r_17541_g.35073_m.35073	VKT7_ANEVI	U-actitoxin-Avd3j OS=Anemonia viridis OX=51769 PE=3 SV=1	2.5E-16	8.85
Sea anemone type 5 potassium channel toxin family	Gene.48662_r_36543_g.48662_m.48662	KV51_METSE	U-metritoxin-Msn2a OS=Metridium senile OX=6116 PE=3 SV=1	2.1E-07	76.57
Scoloptoxin-11 family	Gene.47169_r_33709_g.47169_m.47169	TX14B_SCODE	Scoloptoxin SSD20 (Fragment) OS=Scolopendra dehaani OX=2609776 PE=1 SV=1	1.9E-20	16.02
Hemostasis impairing toxin					
Snaclec family	Gene.46142_r_31919_g.46142_m.46142	SLB9_MACLB	Snaclec B9 OS=Macrovipera lebetina OX=8709 PE=2 SV=1	6.1E-09	8.78

	Gene.36055_r_18475_g.36055_m.36055	SL3_SISCA	Snaclec 3 OS=Sistrurus catenatus edwardsii OX=8762 PE=2 SV=1	4.1E-11	356.96
SNTX/VTX toxin family	Gene.34675_r_17159_g.34675_m.34675	CTXB_SCOPL	Cytolytic toxin-beta OS=Scorpaena plumieri OX=274700 PE=1 SV=1	1.9E-21	8.53
			Other toxins		
AVIT (prokineticin) family	Gene.36658_r_19081_g.36658_m.36658	VP164_LYCMC	Venom protein 164 OS=Lychas mucronatus OX=172552 PE=3 SV=1	3.9E-06	6.71
	Gene.28814_r_12460_g.28814_m.28814	BM8D_BOMMX	Prokineticin Bm8-d OS=Bombina maxima OX=161274 PE=2 SV=1	1.6E-06	19.34
Conocap-like	Gene.27537_r_11591_g.27537_m.27537	CCAP_CONVL	Conocap OS=Conus villepinii OX=257347 GN=conocap PE=1 SV=1	1.1E-06	4.01
**Endothelin/sarafotoxin family**	**Gene.8413_r_2134_g.8413_m.8413**	**SRTX_ATREN**	**Sarafotoxinos=Atractaspis engaddensis OX=1343144 PE=1 SV=2**	**8.5E-09**	**53.05**
IGFBP N-terminal domain, Kazal-like, Ig-like C2-type	Gene.26911_r_11196_g.26911_m.26911	IGFBP_CUPSA	Insulin-like growth factor-binding protein-related protein 1 OS=Cupiennius salei OX=6928 PE=2 SV=1	1.1E-60	281.21
PDGF/VEGF growth factor family	Gene.10036_r_2731_g.10036_m.10036	VEGFA_BITGA	Vascular endothelial growth factor A OS=Bitis gabonica OX=8694 PE=1 SV=1	1.2E-08	32.39
Venom complement C3 homolog family	Gene.48677_r_36574_g.48677_m.48677	VCO3_OPHHA	Ophiophagus venom factor OS=Ophiophagus hannah OX=8665 PE=1 SV=1	5.6E-15	12.49

Table S5. Toxin transcripts of *Baseodiscus unicolor*. Unique toxins families are shown in bold.

**Protein family**	**Transcript ID**	**Uniprot accession**	**Description**	**E-value**	**Expression, TPM**
			Pore-forming toxins		

MACPF domain	Gene.67601_B_unicolor_11432_g.67601_m.67601	PV21_POMMA	Perivitellin-2 67 kda subunit OS=Pomacea maculata OX=1245466 PE=1 SV=1	5.6E-90	12.26
Enzymes					
Arthropod phospholipase D family. Class II subfamily	Gene.134482_B_unicolor_60655_g.134482_m.134482	A411_LOXLA	Dermonecrotic toxin llsictox-alphaiv1i (Fragment) OS=Loxosceles laeta OX=58217 PE=2 SV=1	5.5E-23	2.46
	Gene.121778_B_unicolor_43745_g.121778_m.121778	A4A1_LOXIN	Dermonecrotic toxin lisictox-alphaiva1 OS=Loxosceles intermedia OX=58218 PE=1 SV=1	1E-24	22.20
	Gene.105966_B_unicolor_29654_g.105966_m.105966	A51_LOXIN	Dermonecrotic toxin lisictox-alphav1 OS=Loxosceles intermedia OX=58218 PE=1 SV=1	6.5E-45	143.78
Dnase II family	Gene.74427_B_unicolor_13659_g.74427_m.74427	DNA2_ACAPL	Plancitoxin-1 OS=Acanthaster planci OX=133434 PE=1 SV=1	2.5E-89	3.00
Flavin monoamine oxidase family. FIG1 subfamily	Gene.49406_B_unicolor_6706_g.49406_m.49406	OXLA_VIPAA	L-amino-acid oxidase OS=Vipera ammodytes ammodytes OX=8705 PE=1 SV=1	2.2E-44	5.69
Natterin family	Gene.90302_B_unicolor_20347_g.90302_m.90302	NATT3_THANI	Natterin-3 OS=Thalassophryne nattereri OX=289382 PE=1 SV=1	3.9E-0.6	1.84
Phospholipase A2 family	Gene.124081_B_unicolor_46290_g.124081_m.124081	PA21_SCODE	Phospholipase A2 SSD387 OS=Scolopendra dehaani OX=2609776 PE=1 SV=1	2.3E-23	8.12
Phospholipase A2 family. Group II subfamily. D49 sub-subfamily	Gene.155914_B_unicolor_110324_g.155914_m.155914	PA2B_VIPAZ	Basic phospholipase A2 B chain OS=Vipera aspis zinnikeri OX=55427 PE=1 SV=1	6.6E-16	1.36

Phospholipase A2 family. Group III subfamily	Gene.67828_B_unicolor_11496_g.67828_m.67828	PA23_HELSU	Phospholipase A2 isozymes PA3A/PA3B/PA5 OS=Heloderma suspectum OX=8554 PE=1 SV=3	1.8E-34	5.25
Phospholipase B-like family	Gene.41256_B_unicolor_5044_g.41256_m.41256	PLB_DRYCN	Phospholipase-B 81 OS=Drysdalia coronoides OX=66186 PE=1 SV=1	0	1.32
Proteinase inhibitors					
Venom Kunitz-type family	Gene.116042_B_unicolor_37940_g.116042_m.116042	VKT1B_CONCL	Kunitz-type serine protease inhibitor conotoxin Cal9.1b (Fragment) OS=Californiconus californicus OX=1736779 PE=2 SV=1	5.5E-17	9.08
	Gene.112285_B_unicolor_34627_g.112285_m.112285	VKT1_WALAE	Protease inhibitor 1 OS=Walterinnesia aegyptia OX=64182 PE=1 SV=1	2.2E-14	28.02
	Gene.128801_B_unicolor_52271_g.128801_m.128801	VKT_BOMTE	Kunitz-type serine protease inhibitor Bt-KTI OS=Bombus terrestris OX=30195 PE=2 SV=1	3.2E-16	11.22
	Gene.135598_B_unicolor_62534_g.135598_m.135598	VKT1A_CONCL	Kunitz-type serine protease inhibitor conotoxin Cal9.1a OS=Californiconus californicus OX=1736779 PE=2 SV=1	5.8E-16	87.26
	Gene.67501_B_unicolor_11401_g.67501_m.67501	VKT2_BITGA	Kunitz-type serine protease inhibitor bitisilin-2 OS=Bitis gabonica OX=8694 PE=1 SV=1	2.7E-14	31.10
	Gene.134404_B_unicolor_60534_g.134404_m.134404	VKT1C_CONCL	Kunitz-type serine protease inhibitor conotoxin Cal9.1c (Fragment) OS=Californiconus californicus OX=1736779 PE=2 SV=1	3.5E-17	10.94

Venom Kunitz-type family. Sea anemone type 2 potassium channel toxin subfamily	Gene.110156_B_unicolor_32810_g.110156_m.110156	ATPI1_ACTTE	Actinia tenebrosa protease inhibitors OS=Actinia tenebrosa OX=6105 PE=1 SV=1	3.3E-48	129.66
Ion channel inhibitor activity					
CRISP family	Gene.68366_B_unicolor_11667_g.68366_m.68366	CRVP_LATSE	Cysteine-rich venom protein latisemin OS=Laticauda semifasciata OX=8631 PE=2 SV=1	6.6E-26	0.82
	Gene.48770_B_unicolor_6564_g.48770_m.48770	CRVP_OPHHA	Cysteine-rich venom protein ophanin OS=Ophiophagus hannah OX=8665 PE=1 SV=1	2.9E-18	2.02
CRISP family. Venom allergen 5-like subfamily	Gene.53702_B_unicolor_7686_g.53702_m.53702	VA576_SCODE	Scoloptoxin SSD976 OS=Scolopendra dehaani OX=2609776 PE=1 SV=1	2.8E-27	2.07
Scoloptoxin-11 family	Gene.59910_B_unicolor_9247_g.59910_m.59910	TX14B_SCODE	Scoloptoxin SSD20 (Fragment) OS=Scolopendra dehaani OX=2609776 PE=1 SV=1	1.6E-11	1.39
	Gene.75161_B_unicolor_13935_g.75161_m.75161	TXB3A_SCODE	U-scoloptoxin(11)-Ssd3a OS=Scolopendra dehaani OX=2609776 PE=1 SV=1	6.2E-06	2.85
Hemostasis impairing toxin					
Snaclec family	Gene.158425_B_unicolor_118108_g.158425_m.158425	SL6_DABSI	Snaclec 6 OS=Daboia siamensis OX=343250 PE=2 SV=1	2.2E-09	1.90
	Gene.148898_B_unicolor_90567_g.148898_m.148898	SLRB_BITRH	Snaclec rhinocetin subunit beta OS=Bitis rhinoceros OX=715877 PE=1 SV=1	5.7E-12	1.02
	Gene.72526_B_unicolor_13001_g.72526_m.72526	SLAB_BOTJA	Snaclec bothrojaracin subunit beta OS=Bothrops jararaca OX=8724 PE=1 SV=1	4.3E-10	3.63

SNTX/VTX toxin family	Gene.30482_B_uni-color_3180_g.30482_m.30482	STXA_SYNHO	Stonustoxin subunit alpha OS=Synanceia horrida OX=13279 PE=1 SV=3	6.2E-87	1.31
	Gene.132857_B_uni-color_58073_g.132857_m.132857	STXB_SYNVE	Neoverrucotoxin subunit beta OS=Synanceia verrucosa OX=51996 PE=1 SV=1	4.9E-53	0.95
True venom lectin family	Gene.124185_B_uni-color_46399_g.124185_m.124185	LECM1_HYDHA	C-type lectin 1 OS=Hydrophis hardwickii OX=8781 PE=2 SV=1	1.1E-11	1.15
Other toxins candidates					
IGFBP N-terminal domain, Kazal-like, Ig-like C2-type	Gene.73465_B_uni-color_13314_g.73465_m.73465	IGFBP_CUPSA	Insulin-like growth factor-binding protein-related protein 1 OS=Cupiennius salei OX=6928 PE=2 SV=1	2E-56	47.56
**Insulin-like growth factor binding**	**Gene.133104_B_uni-color_58427_g.133104_m.133104**	**VP302_LYCMC**	**Venom protein 302 OS=Lychas mucronatus OX=172552 PE=2 SV=1**	**1.2E-12**	**1.74**
Glycoprotein hormones subunit alpha family	Gene.161711_B_uni-color_129356_g.161711_m.161711	CTHA2_CONVC	Thyrostimulin alpha-2 subunit OS=Conus victoriae OX=319920 PE=1 SV=1	9.3E-12	2.69
LDL-receptor class A domain	Gene.79949_B_uni-color_15712_g.79949_m.79949	CPROH_CONVC	Neuropeptide prohormone-4 OS=Conus victoriae OX=319920 PE=1 SV=1	4.1E-24	15.45

Table S6. Toxin transcripts of *Hubrechtella ijimai*. Unique toxins families are shown in bold.

Protein family	Transcript ID	Uniprot accession	Description	E-value	Expression, TPM
Neurotoxins					
Conopeptide P-like superfamily	Gene.85849_h_ijimai_63935_g.85849_m.85849	TU92_POLAB	Turripeptide Pal9.2 OS=Polystira albida OX=394106 PE=2 SV=1	5.3E-10	1.66
Pore-forming toxins					

Actinoporin family. Conoidea subfamily	Gene.48764_h_ijimai_18798_g.48764_m.48764	ACTP_CONCN	Conoporin-Cn1 OS=Conus consors OX=101297 PE=1 SV=1	4.7E-06	4.42
Cytotoxin A-III - like	Gene.57253_h_ijimai_26396_g.57253_m.57253	CXA3_CERLA	Cytotoxin A-III OS=Cerebratulus lacteus OX=6221 PE=1 SV=1	4.9E-09	9.23
MACPF domain	Gene.16805_h_ijimai_3077_g.16805_m.16805	PV21_POMMA	Perivitellin-2 67 kda subunit OS=Pomacea maculata OX=1245466 PE=1 SV=1	3E-74	8.88
			Enzymes		
Arthropod phospholipase D family. Class II subfamily	Gene.38018_h_ijimai_11669_g.38018_m.38018	A1H2_LOXHI	Dermonecrotic toxin lhsictox-alphaia1ii (Fragment) OS=Loxosceles hirsuta OX=571525 PE=2 SV=1	1.3E-47	50.05
Arthropod phospholipase D family. Class II subfamily. Class iia sub-subfamily	Gene.53539_h_ijimai_22853_g.53539_m.53539	A51_LOXIN	Dermonecrotic toxin lisictox-alphav1 OS=Loxosceles intermedia OX=58218 PE=1 SV=1	2.7E-14	11.19
	Gene.43821_h_ijimai_15293_g.43821_m.43821	BIB11_SICTE	Dermonecrotic toxin stsictox-betaib1i OS=Sicarius terrosus OX=571544 PE=1 SV=1	2.4E-43	2.04
**Glycosyl hydrolase 37 family**	**Gene.84678_h_ijimai_62109_g.84678_m.84678**	**TREA_PIMHY**	**Trehalase OS=Pimpla hypochondriaca OX=135724 GN=tre1 PE=1 SV=1**	**1.7E-26**	**1.74**
Nucleotide pyrophosphatase/phosphodiesterase family	Gene.42207_h_ijimai_14250_g.42207_m.42207	PDE_NAJAT	Venom phosphodiesterase OS=Naja atra OX=8656 PE=1 SV=1	1E-89	1.64
	Gene.19434_h_ijimai_3830_g.19434_m.19434	PDE2_CROAD	Venom phosphodiesterase 2 OS=Crotalus adamanteus OX=8729 PE=1 SV=1	3.1E-21	2.91
Phospholipase A2 family	Gene.59761_h_ijimai_289	PA2_URTCR	Phospholipase A2 A2-actitoxin-Ucs2a OS=Urticina	5.7E-21	4.81

	83_g.59761_ m.59761		crassicornis OX=45621 PE=1 SV=1		
Phospholipase A2 family. Group I subfamily. D49 sub-subfamily	Gene.67873_ h_ijimai_38275_g.67873_ m.67873	PA2B_BUNCE	Basic phospholipase A2 KPA2 OS=Bungarus caeruleus OX=132961 PE=1 SV=1	3.1E-14	0.81
	Gene.96797_ h_ijimai_81889_g.96797_ m.96797	PA2B1_ACAAN	Basic phospholipase A2 acanthin-1 OS=Acanthophis antarcticus OX=8605 PE=1 SV=1	2.1E-17	1.69
Phospholipase A2 family. Group II subfamily. D49 sub-subfamily	Gene.93238_ h_ijimai_75856_g.93238_ m.93238	PA2B_DABRR	Basic phospholipase A2 RVV-VD OS=Daboia russelii OX=8707 PE=1 SV=1	1.3E-15	1.94
	Gene.40461_ h_ijimai_13161_g.40461_ m.40461	PA2BB_TRIST	Basic phospholipase A2 Ts-G6D49 OS=Trimeresurus stejnegeri OX=39682 PE=1 SV=2	1.4E-19	6.17
Phospholipase A2 family. Group III subfamily	Gene.61429_ h_ijimai_30714_g.61429_ m.61429	PA2A2_HELSU	Acidic phospholipase A2 PA4 OS=Heloderma suspectum OX=8554 PE=1 SV=2	2.4E-30	4.40
	Gene.94293_ h_ijimai_77673_g.94293_ m.94293	HEMI1_HEMLE	Phospholipase A2 hemilipin OS=Hemiscorpius lepturus OX=520031 PE=1 SV=1	3.8E-07	3.24
Venom metalloproteinase (M12B) family. P-III subfamily. P-iiia sub-subfamily	Gene.78870_ h_ijimai_53264_g.78870_ m.78870	VM3_NAJAT	Zinc metalloproteinase-disintegrin-like namp OS=Naja atra OX=8656 PE=2 SV=1	7.8E-10	1.29
Venom metalloproteinase (M12B) family. P-II subfamily. P-iib sub-subfamily	Gene.79950_ h_ijimai_54868_g.79950_ m.79950	VM2AG_GLOHA	Zinc metalloproteinase-disintegrin agkistin OS=Gloydius halys OX=8714 PE=2 SV=1	5.6E-19	2.33
Proteinase inhibitors					

**Serine protease inhibitor-like (TIL domain-containing) family**	**Gene.55378_h_ijimai_24527_g.55378_m.55378**	**ALL6_APICE**	**Venom serine protease inhibitor OS=Apis cerana OX=7461 PE=2 SV=1**	**1.3E-06**	**1.03**
Venom Kunitz-type family	Gene.54210_h_ijimai_23459_g.54210_m.54210	VKT2_NAJNI	Kunitz-type serine protease inhibitor 2 OS=Naja nivea OX=8655 PE=1 SV=1	1.5E-13	1.47
	Gene.39354_h_ijimai_12494_g.39354_m.39354	VKT2_BITGA	Kunitz-type serine protease inhibitor bitisilin-2 OS=Bitis gabonica OX=8694 PE=1 SV=1	9.4E-15	2.65
	Gene.76307_h_ijimai_49575_g.76307_m.76307	VKT1_BITGA	Kunitz-type serine protease inhibitor bitisilin-1 OS=Bitis gabonica OX=8694 PE=1 SV=1	1.5E-19	3.42
Venom Kunitz-type family. Sea anemone type 2 potassium channel toxin subfamily	Gene.46349_h_ijimai_17045_g.46349_m.46349	ATPI1_ACTTE	Actinia tenebrosa protease inhibitors OS=Actinia tenebrosa OX=6105 PE=1 SV=1	4.7E-36	52.76
Ion channel inhibitor activity					
CRISP family	Gene.24894_h_ijimai_5652_g.24894_m.24894	MR30_CONMR	Cysteine-rich venom protein Mr30 OS=Conus marmoreus OX=42752 PE=1 SV=1	1.9E-34	3.10
	Gene.49571_h_ijimai_19458_g.49571_m.49571	CRVP1_PSEPL	Cysteine-rich venom protein ENH1 OS=Pseudoferania polylepis OX=338839 PE=2 SV=1	9E-39	3.33
	Gene.54175_h_ijimai_23430_g.54175_m.54175	CRVP2_HYDHA	Cysteine-rich venom protein 2 OS=Hydrophis hardwickii OX=8781 PE=2 SV=2	2.6E-19	2.60
	Gene.39233_h_ijimai_12425_g.39233_m.39233	CRVP_AUSSU	Cysteine-rich venom protein OS=Austrelaps superbus OX=29156 PE=2 SV=1	1E-18	15.81

	Gene.33916_h_ijimai_9465_g.33916_m.33916	CRVP_LATSE	Cysteine-rich venom protein latisemin OS=Laticauda semifasciata OX=8631 PE=2 SV=1	6.3E-19	3.17
	Gene.48497_h_ijimai_18587_g.48497_m.48497	TX31_CONTE	Cysteine-rich venom protein OS=Conus textile OX=6494 PE=1 SV=1	1.8E-23	2.45
CRISP family. Venom allergen 5-like subfamily	Gene.41400_h_ijimai_13775_g.41400_m.41400	VA576_SCODE	Scoloptoxin SSD976 OS=Scolopendra dehaani OX=2609776 PE=1 SV=1	1.3E-26	22.85
	Gene.78783_h_ijimai_53131_g.78783_m.78783	VA5_TITSE	Venom allergen 5 (Fragments) OS=Tityus serrulatus OX=6887 PE=1 SV=1	1.4E-07	0.77
Hemostasis impairing toxin					
Snaclec family	Gene.76159_h_ijimai_49380_g.76159_m.76159	SL124_ECHOC	Snaclec CTL-Eoc124 OS=Echis ocellatus OX=99586 PE=2 SV=1	3.5E-06	0.56
	Gene.93937_h_ijimai_77005_g.93937_m.93937	SLAD_MACLB	Snaclec A13 OS=Macrovipera lebetina OX=8709 PE=2 SV=1	2E-08	1.73
	Gene.87733_h_ijimai_66957_g.87733_m.87733	SLA_PROFL	Snaclec coagulation factor IX-binding protein subunit A OS=Protobothrops flavoviridis OX=88087 PE=1 SV=1	3.1E-09	0.82
	Gene.54259_h_ijimai_23503_g.54259_m.54259	SLBB_TRIAB	Snaclec alboaggregin-B subunit beta OS=Trimeresurus albolabris OX=8765 PE=1 SV=2	1.7E-11	0.60
SNTX/VTX toxin family	Gene.15901_h_ijimai_2845_g.15901_m.15901	STXB_SYNVE	Neoverrucotoxin subunit beta OS=Synanceia verrucosa OX=51996 PE=1 SV=1	2.1E-96	3.48
	Gene.62213_h_ijimai_31569_g.62213_m.62213	CTXA_SCOPL	Cytolytic toxin-alpha OS=Scorpaena plumieri	1.6E-31	2.57

			OX=274700 PE=1 SV=1		
True venom lectin family	Gene.88129_h_ijimai_67546_g.88129_m.88129	LEC1_LEIMD	C-type lectin lec-toxin-Lei1 OS=Leioheterodon madagascariensis OX=46577 PE=2 SV=1	2.6E-06	2.10
	Gene.72600_h_ijimai_44478_g.72600_m.72600	LECM1_HYDHA	C-type lectin 1 OS=Hydrophis hardwickii OX=8781 PE=2 SV=1	1.8E-09	0.28
			Other toxins candidates		
AVIT (prokineticin) family	Gene.57892_h_ijimai_27009_g.57892_m.57892	BM8E_BOMMX	Prokineticin Bm8-e OS=Bombina maxima OX=161274 PE=2 SV=1	2.9E-06	6.50
EGF-like, Sushi, TSP type-1, LDL-receptor class A	Gene.62342_h_ijimai_31711_g.62342_m.62342	CTX_SEPES	SE-cephalotoxin OS=Sepia esculenta OX=31210 PE=1 SV=1	1.8E-28	0.96
IGFBP N-terminal, Kazal-like, Ig-like C2-type	Gene.33729_h_ijimai_9376_g.33729_m.33729	IGFBP_CUPSA	Insulin-like growth factor-binding protein-related protein 1 OS=Cupiennius salei OX=6928 PE=2 SV=1	2.1E-53	24.19

Table S7. Toxin transcripts of *Lineus longissimus*. Unique toxins families are shown in bold.

Protein family	Transcript ID	Uniprot accession	Description	E-value	Expression, TPM
			Neurotoxins		
**Neurotoxin 02 (plectoxin) family. 02 (plectoxin) subfamily.**	**Gene.137391_l_long35194_g.137391_m.137391**	**TX22A_PLETR**	**Omega-plectoxin-Pt1a OS=Plectreurys tristis OX=33319 PE=1 SV=1**	**2.5E-06**	**152.67**
			Pore-forming toxins		
Cytotoxin A-III-like	Gene.136669_l_long34583_g.136669_m.136669	CXA3_CERLA	Cytotoxin A-III OS=Cerebratulus lacteus OX=6221 PE=1 SV=1	2E-45	0.24
MACPF domain	Gene.63359_l_long8572_g.63359_m.63359	PV21_POMMA	Perivitellin-2 67 kda subunit OS=Pomacea	2.4E-89	6.78

			maculata OX=1245466 PE=1 SV=1		
		Enzymes			
Arthropod phospholipase D family. Class II subfamily	Gene.130745_l_long30563_g.130745_m.130745	A1L1_LOXSA	Dermonecrotic toxin lsasictox-alphaib2i (Fragment) OS=Loxosceles sabina OX=571529 PE=2 SV=1	4.7E-35	1.83
Dnase II family	Gene.110707_l_long21360_g.110707_m.110707	DNA2_ACAPL	Plancitoxin-1 OS=Acanthaster planci OX=133434 PE=1 SV=1	4.2E-94	8.26
Flavin monoamine oxidase family. FIG1 subfamily	Gene.48707_l_long5836_g.48707_m.48707	OXLA_CALRH	L-amino-acid oxidase OS=Calloselasma rhodostoma OX=8717 PE=1 SV=2	3E-44	1.90
**Multicopper oxidase family**	**Gene.125889_l_long27832_g.125889_m.125889**	**FA5_PSETE**	**Coagulation factor V OS=Pseudonaja textilis OX=8673 GN=F5 PE=1 SV=1**	**2.5E-24**	**0.59**
Natterin family	Gene.131057_l_long30751_g.131057_m.131057	NATT4_THANI	Natterin-4 OS=Thalassophryne nattereri OX=289382 PE=2 SV=1	1.5E-07	0.80
Phospholipase A2 family. Group I subfamily. D49 sub-subfamily	Gene.107060_l_long20049_g.107060_m.107060	PA2B_MICCO	Basic phospholipase A2 OS=Micrurus corallinus OX=54390 PE=1 SV=1	1.2E-19	15.88
Phospholipase A2 family. Group III subfamily	Gene.139946_l_long37449_g.139946_m.139946	PA2A2_HELSU	Acidic phospholipase A2 PA4 OS=Heloderma suspectum OX=8554 PE=1 SV=2	4.2E-30	0.23
	Gene.100331_l_long17837_g.100331_m.100331	PA23_HELSU	Phospholipase A2 isozymes PA3A/PA3B/PA5 OS=Heloderma suspectum OX=8554 PE=1 SV=3	8.4E-33	17.55
Venom metalloproteinase (M12B) family. P-III	Gene.17811_l_long1502_g.17811_m.17811	VM3_BUNMU	Zinc metalloproteinase-disintegrin-like bmmp OS=Bungarus	1.3E-53	0.42

subfamily. P-iiia sub-subfamily			multicinctus OX=8616 PE=1 SV=1		
Proteinase inhibitors					
**Cystatin family**	**Gene.144061_l_long42022_g.144061_m.144061**	**CYT_CHIGU**	**Cystatin-1 OS=Chilobrachys guangxiensis OX=278060 PE=2 SV=1**	**3.3E-09**	**19.40**
**Kazal-like**	**Gene.141669_l_long39209_g.141669_m.141669**	**IELA_ANESU**	**PI-actitoxin-Avd5a OS=Anemonia sulcata OX=6108 PE=1 SV=1**	**1.6E-09**	**0.97**
Natriuretic peptide family	Gene.101475_l_long18215_g.101475_m.101475	SVMI1_CERCE	Snake venom metalloprotease inhibitor 02A10 OS=Cerastes cerastes OX=8697 GN=Svmpi-Cce12 PE=1 SV=1	4.3E-09	0.29
Venom Kunitz-type family	Gene.147661_l_long47205_g.147661_m.147661	VKT2_BITGA	Kunitz-type serine protease inhibitor bitisilin-2 OS=Bitis gabonica OX=8694 PE=1 SV=1	1.6E-14	0.42
	Gene.143563_l_long41377_g.143563_m.143563	VKT1D_CONCL	Kunitz-type serine protease inhibitor conotoxin Cal9.1d (Fragment) OS=Californiconus californicus OX=1736779 PE=2 SV=1	1.5E-17	21.65
	Gene.138804_l_long36403_g.138804_m.138804	VKT1A_CONCL	Kunitz-type serine protease inhibitor conotoxin Cal9.1a OS=Californiconus californicus OX=1736779 PE=2 SV=1	2.2E-17	13.22
Venom Kunitz-type family. Sea anemone type 2 potassium channel toxin subfamily	Gene.87468_l_long14140_g.87468_m.87468	VKT2H_HETCR	PI-stichotoxin-Hcr2h OS=Heteractis crispa OX=175771 PE=3 SV=1	4.4E-16	1.79
Venom Kunitz-type family. Scorpion delta-Ktx subfamily. Delta-	Gene.127205_l_long28520_g.127205_m.127205	VKT12_MESMA	Kunitz-type serine protease inhibitor bmktt-3 OS=Mesobuthus martensii OX=34649 PE=2 SV=1	6.7E-15	0.78

Ktx 1 sub-sub-family	Gene.140308_l_long37783_g.140308_m.140308	VKT11_HOFGE	Kunitz-type serine protease inhibitor Hg1 OS=Hoffmannihadrurus gertschi OX=380989 PE=1 SV=1	2.6E-15	0.42
Ion channel inhibitor activity					
CRISP family	Gene.157145_l_long67833_g.157145_m.157145	CRVP_VIPBN	Cysteine-rich venom protein (Fragment) OS=Vipera berus nikolskii OX=1808362 PE=2 SV=1	2.6E-13	0.32
	Gene.130943_l_long30684_g.130943_m.130943	CRVP_NOTSC	Cysteine-rich venom protein pseudechetoxin-like OS=Notechis scutatus scutatus OX=70142 PE=2 SV=1	8E-18	0.36
	Gene.142151_l_long39713_g.142151_m.142151	CRIS3_HUMAN	Cysteine-rich secretory protein 3 OS=Homo sapiens OX=9606 GN=CRISP3 PE=1 SV=1	2.2E-30	54.62
	Gene.122600_l_long26276_g.122600_m.122600	CRVP_PSEPO	Cysteine-rich venom protein pseudecin OS=Pseudechis porphyriacus OX=8671 PE=1 SV=1	7.5E-33	10.34
	Gene.73577_l_long10775_g.73577_m.73577	CRVP_AUSSU	Cysteine-rich venom protein OS=Austrelaps superbus OX=29156 PE=2 SV=1	2.8E-36	0.07
	Gene.90326_l_long14917_g.90326_m.90326	CRVP_PSEAU	Cysteine-rich venom protein pseudechetoxin OS=Pseudechis australis OX=8670 PE=1 SV=1	8.9E-38	1.61
	Gene.121444_l_long25753_g.121444_m.121444	MR30_CONMR	Cysteine-rich venom protein Mr30 OS=Conus marmoreus OX=42752 PE=1 SV=1	1.6E-37	0.15

	Gene.125968_l_long27877_g.125968_m.125968	CRVP_LATSE	Cysteine-rich venom protein latisemin OS=Laticauda semifasciata OX=8631 PE=2 SV=1	1.7E-15	6.31
CRISP family. Venom allergen 5-like subfamily	Gene.123301_l_long26609_g.123301_m.123301	VA543_SCODE	Scoloptoxin SSD43 OS=Scolopendra dehaani OX=2609776 PE=1 SV=1	6E-27	0.91
Scoloptoxin-11 family	Gene.119454_l_long24839_g.119454_m.119454	TXB3A_SCODE	U-scoloptoxin(11)-Ssd3a OS=Scolopendra dehaani OX=2609776 PE=1 SV=1	2E-07	21.20
			Hemostasis impairing toxin		
Snaclec family	Gene.141292_l_long38803_g.141292_m.141292	SLCB_DEIAC	Snaclec agkicetin-C subunit beta OS=Deinagkistrodon acutus OX=36307 PE=1 SV=1	4.2E-08	15.18
	Gene.102923_l_long18662_g.102923_m.102923	SLDB_TRIAB	Snaclec alboaggregin-D subunit beta OS=Trimeresurus albolabris OX=8765 PE=1 SV=1	3.6E-10	1.58
	Gene.112660_l_long22103_g.112660_m.112660	SLLC1_MACLB	Snaclec coagulation factor X-activating enzyme light chain 1 OS=Macrovipera lebetina OX=8709 GN=LC1 PE=1 SV=1	1.9E-12	0.88
	Gene.9577_l_long658_g.9577_m.9577	SL3_SISCA	Snaclec 3 OS=Sistrurus catenatus edwardsii OX=8762 PE=2 SV=1	2.4E-13	0.50
	Gene.67592_l_long9457_g.67592_m.67592	SLA_BITAR	Snaclec bitiscetin subunit alpha OS=Bitis arietans OX=8692 PE=1 SV=1	3.3E-14	0.54
True venom lectin family	Gene.146352_l_long45155_g.146352_m.146352	LECG1_BUNMU	C-type lectin BML-1 OS=Bungarus multicinctus OX=8616 PE=2 SV=1	5.1E-09	0.28
	Gene.102764_l_long18613	LEC1_MICCO	C-type lectin OS=Micrurus	7.8E-08	0.72

	_g.102764_m.102764		corallinus OX=54390 PE=1 SV=1		
	Gene.99209_l_long17495_g.99209_m.99209	LECM1_PHIOL	C-type lectin lectoxin-Phi1 OS=Philodryas olfersii OX=120305 PE=2 SV=1	5E-10	0.18
Other toxins candidates					
Conocap - like	Gene.38449_l_long4188_g.38449_m.38449	CCAP_CONVL	Conocap OS=Conus villepinii OX=257347 GN=conocap PE=1 SV=1	8.7E-07	5.49
EGF domain peptide family	Gene.123598_l_long26751_g.123598_m.123598	TX18A_MANRB	U18-myrmicitoxin-Mri1a OS=Manica rubida OX=219785 PE=1 SV=1	7.7E-07	1.34
Glycoprotein hormones subunit alpha family	Gene.117298_l_long23931_g.117298_m.117298	CTHA2_CONVC	Thyrostimulin alpha-2 subunit OS=Conus victoriae OX=319920 PE=1 SV=1	7.4E-19	7.02
Glycoprotein hormones subunit beta family	Gene.120462_l_long25319_g.120462_m.120462	CTHB5_CONVC	Thyrostimulin beta-5 subunit OS=Conus victoriae OX=319920 PE=2 SV=1	9.9E-19	11.19
IGFBP N-terminal domain	Gene.151742_l_long54638_g.151742_m.151742	VP302_LYCMC	Venom protein 302 OS=Lychas mucronatus OX=172552 PE=2 SV=1	1.8E-08	0.40
IGFBP N-terminal, Kazal-like, Ig-like C2-type	Gene.129083_l_long29578_g.129083_m.129083	IGFBP_CUPSA	Insulin-like growth factor-binding protein-related protein 1 OS=Cupiennius salei OX=6928 PE=2 SV=1	6.2E-52	9.67
Insulin family	Gene.100267_l_long17817_g.100267_m.100267	INS2C_CONFO	Con-Ins F2c OS=Conus floridulus OX=97180 PE=1 SV=1	9.3E-09	2.85
	Gene.109882_l_long21067_g.109882_m.109882	INS1_CONIM	Con-Ins Im1 OS=Conus imperialis OX=35631 PE=2 SV=1	2.6E-14	1.25
LDL-receptor class A domain	Gene.99182_l_long17485_g.99182_m.99182	CPROH_CONVC	Neuropeptide prohormone-4 OS=Conus victoriae OX=319920 PE=1 SV=1	8.6E-24	11.07

NGF-beta family	Gene.71709_l_long10348_g.71709_m.71709	NGFV1_PSEAU	Venom nerve growth factor 1 OS=Pseudechis australis OX=8670 PE=2 SV=1	1.8E-12	0.43

Table S8. Toxin transcripts of *Lineus ruber*.

Protein family	Transcript ID	Uniprot accession	Description	E-value	Expression, TPM
		Pore-forming toxins			
Actinoporin family. Conoidea subfamily	Gene.59713_L_ruber_19170_g.59713_m.59713	ACTP1_TERAN	Tereporin-Ca1 (Fragment) OS=Terebra anilis OX=553697 PE=2 SV=1	2.1E-28	45.91
Cytotoxin A-III-like	Gene.88551_L_ruber_53287_g.88551_m.88551	CXA3_CERLA	Cytotoxin A-III OS=Cerebratulus lacteus OX=6221 PE=1 SV=1	7.9E-42	63.10
MACPF domain	Gene.29509_L_ruber_5163_g.29509_m.29509	PV21_POMMA	Perivitellin-2 67 kda subunit OS=Pomacea maculata OX=1245466 PE=1 SV=1	3.9E-92	3.01
		Enzymes			
Arthropod phospholipase D family. Class II subfamily	Gene.50684_L_ruber_13690_g.50684_m.50684	A1LB1_LOXAR	Dermonecrotic toxin larsictox-alphaib2bi (Fragment) OS=Loxosceles arizonica OX=196454 PE=1 SV=1	1.6E-29	7.57
Arthropod phospholipase D family. Class II subfamily. Class iia sub-subfamily	Gene.40924_L_ruber_9156_g.40924_m.40924	A4A1_LOXIN	Dermonecrotic toxin lisictox-alphaiva1 OS=Loxosceles intermedia OX=58218 PE=1 SV=1	1.7E-39	17.86
Arthropod phospholipase D family. Class II subfamily. Class iib sub-subfamily	Gene.95693_L_ruber_66547_g.95693_m.95693	B1Q_LOXIN	Dermonecrotic toxin lisictox-betaid1 OS=Loxosceles intermedia OX=58218 PE=1 SV=1	3.1E-13	0.50
	Gene.89353_L_ruber_547	B1H_LOXLA	Dermonecrotic toxin llsictox-	7E-22	0.51

	04_g.89353_m.89353		betaia1 OS=Loxosceles laeta OX=58217 PE=1 SV=1		
	Gene.41631_L_ruber_9450_g.41631_m.41631	B1HA_LOXBO	Dermonecrotic toxin lbsictox-betaia1a OS=Loxosceles boneti OX=283164 PE=1 SV=2	2.1E-41	6.18
Dnase II family	Gene.48438_L_ruber_12571_g.48438_m.48438	DNA2_ACAPL	Plancitoxin-1 OS=Acanthaster planci OX=133434 PE=1 SV=1	1.9E-91	0.98
Flavin monoamine oxidase family. FIG1 subfamily	Gene.23011_L_ruber_3399_g.23011_m.23011	OXLA_BOTAT	L-amino-acid oxidase OS=Bothrops atrox OX=8725 PE=1 SV=2	5.9E-48	1.84
Phospholipase A2 family. Group I subfamily. D49 sub-subfamily	Gene.45934_L_ruber_11378_g.45934_m.45934	PA2B_MICCO	Basic phospholipase A2 OS=Micrurus corallinus OX=54390 PE=1 SV=1	5E-20	4.17
Phospholipase A2 family. Group III subfamily	Gene.53961_L_ruber_15534_g.53961_m.53961	PA23_HELSU	Phospholipase A2 isozymes PA3A/PA3B/PA5 OS=Heloderma suspectum OX=8554 PE=1 SV=3	6.8E-30	7.76
	Gene.36510_L_ruber_7471_g.36510_m.36510	PA2A2_HELSU	Acidic phospholipase A2 PA4 OS=Heloderma suspectum OX=8554 PE=1 SV=2	1.5E-32	2.39
Proteinase inhibitors					
Venom Kunitz-type family. Scorpion delta-Ktx subfamily. Delta-Ktx 3 sub-subfamily	Gene.54609_L_ruber_15904_g.54609_m.54609	VKT31_MESMA	Kunitz-type serine protease inhibitor bmktt-2 OS=Mesobuthus martensii OX=34649 PE=2 SV=2	2.2E-15	35.16
Ion channel inhibitor activity					
CRISP family	Gene.48539_L_ruber_12613_g.48539_m.48539	CRVP_LATSE	Cysteine-rich venom protein latisemin OS=Laticauda semifasciata OX=8631 PE=2 SV=1	4.9E-23	2.40

	Gene.70348_L_ruber_28288_g.70348_m.70348	GPRL1_BOVIN	GLIPR1-like protein 1 OS=Bos taurus OX=9913 GN=GLIPR1L1 PE=1 SV=1	2.4E-25	6.97
	Gene.48775_L_ruber_12726_g.48775_m.48775	CRVP_AUSSU	Cysteine-rich venom protein OS=Austrelaps superbus OX=29156 PE=2 SV=1	1.2E-28	2.48
	Gene.45105_L_ruber_10968_g.45105_m.45105	CRVP_ERYPO	Cysteine-rich venom protein LIO1 (Fragment) OS=Erythrolamprus poecilogyrus OX=338838 PE=2 SV=1	2.5E-30	1.61
	Gene.47888_L_ruber_12310_g.47888_m.47888	CRVP_PHIOL	Cysteine-rich venom protein OS=Philodryas olfersii OX=120305 PE=2 SV=1	2.7E-31	2.66
	Gene.50154_L_ruber_13416_g.50154_m.50154	MR30_CONMR	Cysteine-rich venom protein Mr30 OS=Conus marmoreus OX=42752 PE=1 SV=1	6.9E-38	2.03
	Gene.57217_L_ruber_17556_g.57217_m.57217	CRVP_PSEAU	Cysteine-rich venom protein pseudechetoxin OS=Pseudechis australis OX=8670 PE=1 SV=1	4.8E-40	0.44
CRISP family. Venom allergen 5-like subfamily	Gene.42835_L_ruber_9947_g.42835_m.42835	VA576_SCODE	Scoloptoxin SSD976 OS=Scolopendra dehaani OX=2609776 PE=1 SV=1	1.1E-32	1.62
Nemertide family	Gene.99712_L_ruber_74618_g.99712_m.99712	NEMA1_LINLO	Nemertide alpha-1 (Fragment) OS=Lineus longissimus OX=88925 PE=1 SV=1	1.4E-35	20.54
Venom Kunitz-type family. Sea anemone type 2 potassium channel toxin subfamily	Gene.87032_L_ruber_50599_g.87032_m.87032	VKT5_ANEVI	U-actitoxin-Avd3h OS=Anemonia viridis OX=51769 PE=3 SV=1	4.1E-16	140.87
Hemostasis impairing toxin					

Snaclec family	Gene.52300_L_ruber_14563_g.52300_m.52300	SL3_SISCA	Snaclec 3 OS=Sistrurus catenatus edwardsii OX=8762 PE=2 SV=1	3.6E-13	1.74
	Gene.50378_L_ruber_13534_g.50378_m.50378	SLA_BITAR	Snaclec bitiscetin subunit alpha OS=Bitis arietans OX=8692 PE=1 SV=1	4.7E-15	0.00
SNTX/VTX toxin family	Gene.19632_L_ruber_2633_g.19632_m.19632	STXB_SYNVE	Neoverrucotoxin subunit beta OS=Synanceia verrucosa OX=51996 PE=1 SV=1	2.5E-99	0.88
True venom lectin family	Gene.82960_L_ruber_44078_g.82960_m.82960	LECM1_PHIOL	C-type lectin lectoxin-Phi1 OS=Philodryas olfersii OX=120305 PE=2 SV=1	1.9E-15	0.53
Other toxins candidates					
Glycoprotein hormones subunit alpha family	Gene.62977_L_ruber_21677_g.62977_m.62977	CTHA2_CONVC	Thyrostimulin alpha-2 subunit OS=Conus victoriae OX=319920 PE=1 SV=1	6.6E-20	0.24
Glycoprotein hormones subunit beta family	Gene.64507_L_ruber_22903_g.64507_m.64507	CTHB5_CONVC	Thyrostimulin beta-5 subunit OS=Conus victoriae OX=319920 PE=2 SV=1	2.1E-18	0.60
IGFBP N-terminal, Kazal-like, Ig-like C2-type	Gene.22728_L_ruber_3330_g.22728_m.22728	IGFBP_CUPSA	Insulin-like growth factor-binding protein-related protein 1 OS=Cupiennius salei OX=6928 PE=2 SV=1	1.3E-51	3.45
LDL-receptor class A domain	Gene.29081_L_ruber_5035_g.29081_m.29081	CPROH_CONVC	Neuropeptide prohormone-4 OS=Conus victoriae OX=319920 PE=1 SV=1	1.7E-24	8.09
Venom complement C3 homolog family	Gene.80447_L_ruber_40451_g.80447_m.80447	VCO3_OPHHA	Ophiophagus venom factor OS=Ophiophagus hannah OX=8665 PE=1 SV=1	3E-19	1.52

Table S9. Toxin transcripts of *Lineus sanguineus*. Unique toxins families are shown in bold.

Protein family	Transcript ID	Uniprot accession	Description	E-value	Expression, TPM
			Neurotoxins		
Conopeptide P-like superfamily	Gene.81947_L_sanguineus_50972_g.81947_m.81947	TU91_LOPOL	Turripeptide Lol9.1 OS=Lophiotoma olangoensis OX=2420066 PE=2 SV=1	2.5E-07	1.52
	Gene.86098_L_sanguineus_56633_g.86098_m.86098	TU11_LOPAL	Turripeptide OL11-like (Fragment) OS=Lophiotoma albina OX=1525239 PE=2 SV=1	3.1E-08	1.41
**Neurotoxin 04 (omega-agtx) family. 02 (Tx1) subfamily**	**Gene.68419_L_sanguineus_35394_g.68419_m.68419**	**TXL1_PHONI**	**Mu-ctenitoxin-Pn1a OS=Phoneutria nigriventer OX=6918 PE=1 SV=2**	**4.6E-06**	**185.65**
**Neurotoxin 10 (Hwtx-1) family. 15 (Hntx-3) subfamily**	**Gene.74066_L_sanguineus_41516_g.74066_m.74066**	**H3A08_CYRHA**	**Hainantoxin-III 8 OS=Cyriopagopus hainanus OX=209901 PE=1 SV=1**	**1.2E-07**	**564.98**
			Pore-forming toxins		
Cytotoxin A-III-like	Gene.52528_L_sanguineus_21731_g.52528_m.52528	CXA3_CERLA	Cytotoxin A-III OS=Cerebratulus lacteus OX=6221 PE=1 SV=1	2.9E-47	427.35
MACPF domain	Gene.82474_L_sanguineus_51662_g.82474_m.82474	PV21_POMCA	Perivitellin-2 67 kda subunit OS=Pomacea canaliculata OX=400727 PE=1 SV=2	2.6E-15	1.38
	Gene.61455_L_sanguineus_28889_g.61455_m.61455	PV21_POMMA	Perivitellin-2 67 kda subunit OS=Pomacea maculata OX=1245466 PE=1 SV=1	1.1E-24	2.77
			Enzymes		
Arthropod phospholipase D family. Class II subfamily	Gene.94336_L_sanguineus_69330_g.94336_m.94336	A1LB1_LOXAR	Dermonecrotic toxin larsictox-alphaib2bi (Fragment) OS=Loxosceles arizonica OX=196454 PE=1 SV=1	8.9E-07	1.44
	Gene.55261_L_sanguineus_237	A1OC_LOXRU	Dermonecrotic toxin lrusictox-alphaic1c (Fragment)	1.9E-18	2.30

	98_g.55261_ m.55261		OS=Loxosceles rufescens OX=571528 PE=2 SV=1		
	Gene.40396_ L_sanguineus_14037_g.40396_ m.40396	A1IB1_LOXIN	Dermonecrotic toxin lisictox-alphaia2bi OS=Loxosceles intermedia OX=58218 PE=2 SV=1	2.4E-38	6.73
	Gene.37457_ L_sanguineus_12469_g.37457_ m.37457	A21_LOXAR	Dermonecrotic toxin larsictox-alphaii1 (Fragment) OS=Loxosceles arizonica OX=196454 PE=2 SV=1	1.8E-47	4.00
Arthropod phospholipase D family. Class II subfamily. Class iia sub-subfamily	Gene.29321_ L_sanguineus_8619_g.29321_m. 29321	A51_LOXIN	Dermonecrotic toxin lisictox-alphav1 OS=Loxosceles intermedia OX=58218 PE=1 SV=1	4.3E-46	10.15
Dnase II family	Gene.59918_ L_sanguineus_27583_g.59918_ m.59918	DNA2_ACAPL	Plancitoxin-1 OS=Acanthaster planci OX=133434 PE=1 SV=1	6.6E-56	3.10
Flavin monoamine oxidase family. FIG1 subfamily	Gene.16674_ L_sanguineus_3842_g.16674_m. 16674	OXLA_BOTPC	L-amino acid oxidase (Fragment) OS=Bothrops pictus OX=133440 PE=1 SV=1	1.2E-38	9.77
Natterin family	Gene.29078_ L_sanguineus_8518_g.29078_m. 29078	NATT4_THANI	Natterin-4 OS=Thalassophryne nattereri OX=289382 PE=2 SV=1	1.1E-11	3.44
Nucleotide pyrophosphatase/phosphodiesterase family	Gene.16311_ L_sanguineus_3727_g.16311_m. 16311	PDE2_CROAD	Venom phosphodiesterase 2 OS=Crotalus adamanteus OX=8729 PE=1 SV=1	4E-176	3.81
Peptidase M12A domain	Gene.56334_ L_sanguineus_24634_g.56334_ m.56334	VMPA4_LOXLA	Astacin-like metalloprotease toxin 4 (Fragment) OS=Loxosceles laeta OX=58217 PE=2 SV=1	5.9E-08	1.46

Phospholipase A2 family	Gene.14943_L_sanguineus_3301_g.14943_m.14943	PA21_SCODE	Phospholipase A2 SSD387 OS=Scolopendra dehaani OX=2609776 PE=1 SV=1	6.9E-21	11.86
Phospholipase A2 family. Group I subfamily. D49 sub-subfamily	Gene.107532_L_sanguineus_92735_g.107532_m.107532	PA2B_MICCO	Basic phospholipase A2 OS=Micrurus corallinus OX=54390 PE=1 SV=1	2.6E-06	1.07
	Gene.15767_L_sanguineus_3556_g.15767_m.15767	PA2BA_BUNFA	Basic phospholipase A2 10 (Fragment) OS=Bungarus fasciatus OX=8613 PE=1 SV=2	1.6E-20	6.43
Phospholipase A2 family. Group III subfamily	Gene.39305_L_sanguineus_13431_g.39305_m.39305	HEMI1_HEMLE	Phospholipase A2 hemilipin OS=Hemiscorpius lepturus OX=520031 PE=1 SV=1	1.7E-17	29.62
	Gene.35790_L_sanguineus_11591_g.35790_m.35790	PA23_HELSU	Phospholipase A2 isozymes PA3A/PA3B/PA5 OS=Heloderma suspectum OX=8554 PE=1 SV=3	7E-29	5.67
	Gene.27806_L_sanguineus_7962_g.27806_m.27806	PA2A2_HELSU	Acidic phospholipase A2 PA4 OS=Heloderma suspectum OX=8554 PE=1 SV=2	1.2E-33	6.81
Type-B carboxylesterase/lipase family	Gene.23027_L_sanguineus_6063_g.23027_m.23027	ACES_BUNFA	Acetylcholinesterase OS=Bungarus fasciatus OX=8613 GN=ACHE PE=1 SV=2	1.3E-125	2.54
Venom metalloproteinase (M12B) family. P-I subfamily	Gene.78387_L_sanguineus_46574_g.78387_m.78387	VM1B2_BOTJR	Snake venom metalloproteinase bjussump-2 (Fragment) OS=Bothrops jararacussu OX=8726 PE=1 SV=1	7.3E-08	0.61
**Venom metalloproteinase (M12B) family. P-II**	**Gene.86069_L_sanguineus_56583_g.86069_m.86069**	**VM2AB_AGKCO**	**Zinc metalloproteinase/disintegrin OS=Agkistrodon contortrix**	**8.4E-06**	**2.62**

**subfamily. P-iie sub-subfamily**			**contortrix OX=8713 PE=1 SV=1**		
		Proteinase inhibitors			
Venom Kunitz-type family	Gene.82424_L_sanguineus_51597_g.82424_m.82424	VKT_BOMIG	Kunitz-type serine protease inhibitor Bi-KTI OS=Bombus ignitus OX=130704 PE=2 SV=1	2.8E-11	4.18
	Gene.85469_L_sanguineus_55687_g.85469_m.85469	VKT_AUSLA	Putative Kunitz-type serine protease inhibitor OS=Austrelaps labialis OX=471292 PE=2 SV=1	1.6E-12	1.32
	Gene.66438_L_sanguineus_33458_g.66438_m.66438	VKT1A_CONCL	Kunitz-type serine protease inhibitor conotoxin Cal9.1a OS=Californiconus californicus OX=1736779 PE=2 SV=1	1.4E-16	1037.70
	Gene.66701_L_sanguineus_33710_g.66701_m.66701	VKT3_BITGA	Kunitz-type serine protease inhibitor bitisilin-3 (Fragment) OS=Bitis gabonica OX=8694 PE=2 SV=1	1.2E-40	3.14
		Ion channel inhibitor activity			
CRISP family	Gene.50968_L_sanguineus_20637_g.50968_m.50968	CRVP_ERYPO	Cysteine-rich venom protein LIO1 (Fragment) OS=Erythrolamprus poecilogyrus OX=338838 PE=2 SV=1	1.5E-18	1.67
	Gene.35657_L_sanguineus_11530_g.35657_m.35657	CRVP_LATSE	Cysteine-rich venom protein latisemin OS=Laticauda semifasciata OX=8631 PE=2 SV=1	1.9E-23	2.98
	Gene.19374_L_sanguineus_4750_g.19374_m.19374	CRVP_PHIOL	Cysteine-rich venom protein OS=Philodryas olfersii OX=120305 PE=2 SV=1	1.3E-31	4.39
	Gene.45189_L_sanguineus_168	MR30_CONMR	Cysteine-rich venom protein Mr30 OS=Conus	1.9E-40	20.41

	26_g.45189_ m.45189		marmoreus OX=42752 PE=1 SV=1		
CRISP family. Venom allergen 5-like subfamily	Gene.79573_ L_sanguineus_47961_g.79573_ m.79573	VA543_SCODE	Scoloptoxin SSD43 OS=Scolopendra dehaani OX=2609776 PE=1 SV=1	3.3E-10	1.21
	Gene.88376_ L_sanguineus_59969_g.88376_ m.88376	VA5_VESMA	Venom allergen 5 OS=Vespa mandarinia OX=7446 PE=1 SV=1	8.1E-17	0.69
	Gene.68416_ L_sanguineus_35392_g.68416_ m.68416	VA5_POLDO	Venom allergen 5 OS=Polistes dominula OX=743375 PE=1 SV=2	1.1E-19	1.61
	Gene.38598_ L_sanguineus_13043_g.38598_ m.38598	VA576_SCODE	Scoloptoxin SSD976 OS=Scolopendra dehaani OX=2609776 PE=1 SV=1	4.3E-28	33.22
Nemertide family	Gene.54610_ L_sanguineus_23300_g.54610_ m.54610	NEMA1_LINLO	Nemertide alpha-1 (Fragment) OS=Lineus longissimus OX=88925 PE=1 SV=1	1.8E-10	112.85
Hemostasis impairing toxin					
Snaclec family	Gene.97854_ L_sanguineus_75237_g.97854_ m.97854	SLDA_TRIAB	Snaclec alboaggregin-D subunit alpha OS=Trimeresurus albolabris OX=8765 PE=1 SV=1	3.9E-06	0.79
	Gene.92649_ L_sanguineus_66472_g.92649_ m.92649	SL1_SISCA	Snaclec 1 OS=Sistrurus catenatus edwardsii OX=8762 PE=2 SV=1	7.9E-09	2.22
	Gene.90645_ L_sanguineus_63439_g.90645_ m.90645	SLB1_DEIAC	Snaclec agglucetin subunit beta-1 OS=Deinagkistrodon acutus OX=36307 PE=1 SV=1	4.6E-13	0.58
SNTX/VTX toxin family	Gene.97106_ L_sanguineus_74035_g.97106_ m.97106	CTXA_SCOPL	Cytolytic toxin-alpha OS=Scorpaena plumieri OX=274700 PE=1 SV=1	3.1E-13	0.77

	Gene.96616_ L_sanguineus_73187_g.96616_ m.96616	CTXB_SCOPL	Cytolytic toxin-beta OS=Scorpaena plumieri OX=274700 PE=1 SV=1	3.5E-16	0.68
True venom lectin family	Gene.69521_ L_sanguineus_36592_g.69521_ m.69521	LECM_THRJA	C-type lectin lectoxin-Thr1 OS=Thrasops jacksonii OX=186611 PE=2 SV=1	3.9E-08	1.31
			Other toxins candidates		
Glycoprotein hormones subunit alpha family	Gene.62301_ L_sanguineus_29640_g.62301_ m.62301	CTHA2_CONVC	Thyrostimulin alpha-2 subunit OS=Conus victoriae OX=319920 PE=1 SV=1	1.9E-11	1.66
EGF domain peptide family	Gene.42600_ L_sanguineus_15290_g.42600_ m.42600	TX18A_MANRB	U18-myrmicitoxin-Mri1a OS=Manica rubida OX=219785 PE=1 SV=1	4E-09	2.55
EGF-like, Sushi, TSP type-1, LDL-receptor class A	Gene.17262_ L_sanguineus_4049_g.17262_m. 17262	CTX_SEPES	SE-cephalotoxin OS=Sepia esculenta OX=31210 PE=1 SV=1	1.9E-97	3.62
IGFBP N-terminal, Kazal-like, Ig-like C2-type	Gene.31441_ L_sanguineus_9535_g.31441_m. 31441	IGFBP_CUPSA	Insulin-like growth factor-binding protein-related protein 1 OS=Cupiennius salei OX=6928 PE=2 SV=1	2.2E-52	3.46
Insulin family	Gene.87792_ L_sanguineus_59134_g.87792_ m.87792	INS1_CONIM	Con-Ins Im1 OS=Conus imperialis OX=35631 PE=2 SV=1	2.8E-06	1.14
LDL-receptor class A domain	Gene.18530_ L_sanguineus_4462_g.18530_m. 18530	CPROH_CONVC	Neuropeptide prohormone-4 OS=Conus victoriae OX=319920 PE=1 SV=1	6.4E-23	11.06
**Thyroglobulin_1, 2 hits DOMAIN**	**Gene.64710_ L_sanguineus_31821_g.64710_ m.64710**	**PN16_PHONI**	**U24-ctenitoxin-Pn1a OS=Phoneutria nigriventer OX=6918 PE=1 SV=1**	**6.6E-07**	**25.79**

Table S10. Toxin transcripts of *Notospermus geniculatus*. Unique toxins families are shown in bold.

Protein family	Transcript ID	Uniprot accession	Description	E-value	Expression, TPM
			Pore-forming toxins		
Cytotoxin A-III-like	Gene.128743_N_geniculatus_37710_g.128743_m.128743	CXA3_CERLA	Cytotoxin A-III OS=Cerebratulus lacteus OX=6221 PE=1 SV=1	1.2E-38	204.37
MACPF domain	Gene.146480_N_geniculatus_47623_g.146480_m.146480	PV21_POMCA	Perivitellin-2 67 kda subunit OS=Pomacea canaliculata OX=400727 PE=1 SV=2	6.8E-19	0.32
	Gene.44557_N_geniculatus_6765_g.44557_m.44557	PV21_POMMA	Perivitellin-2 67 kda subunit OS=Pomacea maculata OX=1245466 PE=1 SV=1	7.8E-89	6.82
			Enzymes		
Arthropod phospholipase D family. Class II subfamily	Gene.147654_N_geniculatus_48338_g.147654_m.147654	B1O_LOXCS	Dermonecrotic toxin lcssictox-betaic1 (Fragment) OS=Loxosceles cf. Spinulosa (strain GJB-2008) OX=575952 PE=2 SV=1	1.9E-18	0.33
	Gene.73013_N_geniculatus_14545_g.73013_m.73013	A1O_LOXGA	Dermonecrotic toxin lgsictox-alphaic1 OS=Loxosceles gaucho OX=58216 PE=1 SV=1	5E-30	0.24
	Gene.74953_N_geniculatus_15169_g.74953_m.74953	B1T1_LOXSN	Dermonecrotic toxin lspisictox-betaie3i (Fragment) OS=Loxosceles spinulosa OX=571532 PE=2 SV=1	2.2E-38	0.17
Arthropod phospholipase D family. Class II subfamily. Class iia sub-subfamily	Gene.92607_N_geniculatus_21414_g.92607_m.92607	A51_LOXIN	Dermonecrotic toxin lisictox-alphav1 OS=Loxosceles intermedia OX=58218 PE=1 SV=1	2.3E-32	3.53
Arthropod phospholipase D family. Class II subfamily. Class iib sub-subfamily	Gene.87640_N_geniculatus_19525_g.87640_m.87640	B1Q_LOXIN	Dermonecrotic toxin lisictox-betaid1 OS=Loxosceles intermedia OX=58218 PE=1 SV=1	9.2E-42	2.74

Dnase II family	Gene.108701_N_geniculatus_28112_g.108701_m.108701	DNA2_ACAPL	Plancitoxin-1 OS=Acanthaster planci OX=133434 PE=1 SV=1	3.2E-51	11.18
Glycosyl hydrolase 56 family	Gene.176515_N_geniculatus_67089_g.176515_m.176515	HYAL1_MESMA	Hyaluronidase-1 OS=Mesobuthus martensii OX=34649 PE=1 SV=2	2.3E-13	0.00
	Gene.143755_N_geniculatus_46005_g.143755_m.143755	HYAL_ECHOC	Hyaluronidase OS=Echis ocellatus OX=99586 PE=2 SV=1	1.8E-15	0.00
	Gene.149563_N_geniculatus_49486_g.149563_m.149563	HYAL_CONCN	Hyaluronidase conohyal-Cn1 OS=Conus consors OX=101297 PE=1 SV=1	9.3E-17	8.60
	Gene.88765_N_geniculatus_19933_g.88765_m.88765	HYAL_CONPU	Hyaluronidase conohyal-P1 OS=Conus purpurascens OX=41690 PE=1 SV=1	2.1E-40	0.21
	Gene.101095_N_geniculatus_24787_g.101095_m.101095	HUGA_APIME	Hyaluronidase OS=Apis mellifera OX=7460 PE=1 SV=1	3.7E-60	0.00
Flavin monoamine oxidase family. FIG1 subfamily	Gene.46686_N_geniculatus_7256_g.46686_m.46686	OXLA_CALRH	L-amino-acid oxidase OS=Calloselasma rhodostoma OX=8717 PE=1 SV=2	2.1E-46	0.27
Nucleotide pyrophosphatase/phosphodiesterase family	Gene.32725_N_geniculatus_4280_g.32725_m.32725	PDE2_CROAD	Venom phosphodiesterase 2 OS=Crotalus adamanteus OX=8729 PE=1 SV=1	5.2E-129	4.33
Peptidase M12A domain	Gene.125887_N_geniculatus_36255_g.125887_m.125887	VMPA4_LOXLA	Astacin-like metalloprotease toxin 4 (Fragment) OS=Loxosceles laeta OX=58217 PE=2 SV=1	1.6E-06	0.00
**Phospholipase A2 family. Group I subfamily**	**Gene.120949_N_geniculatus_33786_g.**	**PA22_ACAPL**	**Phospholipase A2 AP-PLA2-II OS=Acanthaster**	**4.3E-11**	**0.84**

	**120949_m.120949**		**planci OX=133434 PE=1 SV=1**		
Phospholipase A2 family. Group I subfamily. D49 sub-subfamily	Gene.143594_N_geniculatus_45913_g.143594_m.143594	PA2B_MICCO	Basic phospholipase A2 OS=Micrurus corallinus OX=54390 PE=1 SV=1	2E-12	0.00
Phospholipase A2 family. Group III subfamily	Gene.118636_N_geniculatus_32651_g.118636_m.118636	HEMI1_HEMLE	Phospholipase A2 hemilipin OS=Hemiscorpius lepturus OX=520031 PE=1 SV=1	1.2E-24	2.78
	Gene.72635_N_geniculatus_14421_g.72635_m.72635	PA23_HELSU	Phospholipase A2 isozymes PA3A/PA3B/PA5 OS=Heloderma suspectum OX=8554 PE=1 SV=3	4.5E-37	24.90
Phospholipase A2 family. Group II subfamily. D49 sub-subfamily	Gene.90565_N_geniculatus_20610_g.90565_m.90565	PA2B1_VIPBN	Basic phospholipase A2 chain HDP-1P OS=Vipera berus nikolskii OX=1808362 PE=1 SV=1	1E-22	6.86
Phospholipase A2 family. Group III subfamily	Gene.91289_N_geniculatus_20900_g.91289_m.91289	PA2A2_HELSU	Acidic phospholipase A2 PA4 OS=Heloderma suspectum OX=8554 PE=1 SV=2	3.7E-29	20.81
Phospholipase B-like family	Gene.63302_N_geniculatus_11598_g.63302_m.63302	PLB_DRYCN	Phospholipase-B 81 OS=Drysdalia coronoides OX=66186 PE=1 SV=1	0	11.03
Venom metalloproteinase (M12B) family	Gene.106790_N_geniculatus_27259_g.106790_m.106790	VMP03_EULPE	Venom metalloproteinase 3 OS=Eulophus pennicornis OX=108749 PE=2 SV=1	3.1E-07	2.57
Venom metalloproteinase (M12B) family. P-III subfamily	Gene.137591_N_geniculatus_42492_g.137591_m.137591	VM34_DRYCN	Zinc metalloproteinase-disintegrin-like MTP4 OS=Drysdalia coronoides OX=66186 PE=1 SV=1	1.5E-23	0.00

**Venom metalloproteinase (M12B) family. P-III subfamily. P-iiib subsubfamily**	**Gene.133242_N_geniculatus_40099_g.133242_m.133242**	**VM3HA_PROFL**	**Zinc metalloproteinase/disintegrin-like HR1a OS=Protobothrops flavoviridis OX=88087 PE=1 SV=1**	**4.6E-09**	**0.00**
			Proteinase inhibitors		
Venom Kunitz-type family	Gene.160680_N_geniculatus_56483_g.160680_m.160680	VKTHD_DENAN	Kunitz-type serine protease inhibitor homolog delta-dendrotoxin OS=Dendroaspis angusticeps OX=8618 PE=1 SV=1	2.1E-09	0.00
	Gene.132845_N_geniculatus_39889_g.132845_m.132845	VKT4_PSETT	Kunitz-type serine protease inhibitor textilinin-4 OS=Pseudonaja textilis textilis OX=169397 PE=2 SV=1	3E-09	3.55
	Gene.159195_N_geniculatus_55539_g.159195_m.159195	VKTCT_OPHHA	Kunitz-type serine protease inhibitor TCI OS=Ophiophagus hannah OX=8665 PE=1 SV=1	3.2E-16	6.38
	Gene.123192_N_geniculatus_34877_g.123192_m.123192	VKT1_TRILK	Kunitz-type U19-barytoxin-Tl1a OS=Trittame loki OX=1295018 PE=2 SV=2	8E-16	0.00
	Gene.147622_N_geniculatus_48319_g.147622_m.147622	VKT1_OXYMI	Kunitz-type serine protease inhibitor microlepidin-1 OS=Oxyuranus microlepidotus OX=111177 PE=2 SV=1	1.4E-19	142.80
	Gene.142227_N_geniculatus_45116_g.142227_m.142227	VKT3_BITGA	Kunitz-type serine protease inhibitor bitisilin-3 (Fragment) OS=Bitis gabonica OX=8694 PE=2 SV=1	1.9E-31	32.21
Venom Kunitz-type family. Sea anemone type 2 potassium	Gene.87698_N_geniculatus_19544_g.87698_m.87698	VKT1_ANTAF	PI-actitoxin-Axm2a OS=Anthopleura aff. Xanthogrammica OX=152178 PE=1 SV=1	9.5E-20	0.00

channel toxin subfamily					
		Ion channel inhibitor activity			
**Conotoxin I3 superfamily**	**Gene.158909_N_geniculatus_55362_g.158909_m.158909**	**I3B2_CONPL**	**Conotoxin Pu11.2 OS=Conus pulicarius OX=93154 PE=3 SV=1**	**9.7E-06**	**3.61**
CRISP family	Gene.178605_N_geniculatus_68509_g.178605_m.178605	VA3_DINQU	Venom allergen 3 homolog OS=Dinoponera quadriceps OX=609295 PE=2 SV=1	2.4E-08	0.00
	Gene.43244_N_geniculatus_6474_g.43244_m.43244	CRVP2_DISTY	Cysteine-rich venom protein DIS2 OS=Dispholidus typus OX=46295 PE=2 SV=1	9.2E-08	0.71
	Gene.71706_N_geniculatus_14138_g.71706_m.71706	CRVP_CROAT	Cysteine-rich venom protein catrin OS=Crotalus atrox OX=8730 PE=1 SV=1	1.5E-24	0.16
	Gene.106517_N_geniculatus_27136_g.106517_m.106517	CRVP_OXYMI	Cysteine-rich venom protein pseudechetoxin-like OS=Oxyuranus microlepidotus OX=111177 PE=2 SV=1	3.5E-30	0.87
	Gene.79877_N_geniculatus_16779_g.79877_m.79877	CRVP_PHIOL	Cysteine-rich venom protein OS=Philodryas olfersii OX=120305 PE=2 SV=1	7E-34	2.01
	Gene.109329_N_geniculatus_28397_g.109329_m.109329	CRVP_LEIMD	Cysteine-rich venom protein LEI1 (Fragment) OS=Leioheterodon madagascariensis OX=46577 PE=1 SV=1	1.6E-34	1.99
	Gene.110897_N_geniculatus_29081_g.110897_m.110897	MR30_CONMR	Cysteine-rich venom protein Mr30 OS=Conus marmoreus OX=42752 PE=1 SV=1	2.2E-34	0.91

	Gene.71903_N_geniculatus_14199_g.71903_m.71903	CRVP_PSETE	Cysteine-rich venom protein pseudechetoxin-like OS=Pseudonaja textilis OX=8673 PE=1 SV=1	3.5E-33	0.48
	Gene.92364_N_geniculatus_21318_g.92364_m.92364	TX31_CONTE	Cysteine-rich venom protein OS=Conus textile OX=6494 PE=1 SV=1	7.7E-34	2.06
	Gene.33460_N_geniculatus_4413_g.33460_m.33460	CRVP_AUSSU	Cysteine-rich venom protein OS=Austrelaps superbus OX=29156 PE=2 SV=1	3.2E-34	0.63
CRISP family. Venom allergen 5-like subfamily	Gene.111771_N_geniculatus_29480_g.111771_m.111771	VA5_VESPE	Venom allergen 5 OS=Vespula pensylvanica OX=30213 PE=1 SV=1	4.5E-54	0.00
Sea anemone structural class 9a family	Gene.63263_N_geniculatus_11585_g.63263_m.63263	TX9A_ANTMC	Delta-actitoxin-Amc1a OS=Antheopsis maculata OX=280228 PE=1 SV=1	2.5E-11	19.21
Scoloptoxin-05 family	Gene.93901_N_geniculatus_21904_g.93901_m.93901	TX53A_ETHRU	U-scoloptoxin(05)-Er3a OS=Ethmostigmus rubripes OX=62613 PE=2 SV=1	2.7E-06	0.47
Scoloptoxin-11 family	Gene.109455_N_geniculatus_28449_g.109455_m.109455	TXB2A_SCOAL	U-scoloptoxin(11)-Sa2a OS=Scolopendra alternans OX=1329349 PE=2 SV=1	1.3E-06	2.36
Venom Kunitz-type family. Sea anemone type 2 potassium channel toxin subfamily	Gene.155096_N_geniculatus_52945_g.155096_m.155096	VKT7_ANEVI	U-actitoxin-Avd3j OS=Anemonia viridis OX=51769 PE=3 SV=1	3.7E-19	4.04
Hemostasis impairing toxin					
Snaclec family	Gene.60567_N_geniculatus_10834_g.60567_m.60567	SLA4_TRIAB	Snaclec alboaggregin-A subunit beta' OS=Trimeresurus albolabris OX=8765 PE=1 SV=1	4E-06	1.01
	Gene.161623_N_geniculatus_57108_g.	SLA_BOTIN	Snaclec bothroinsularin subunit alpha OS=Bothrops	1.8E-13	0.00

	161623_m.161623		insularis OX=8723 PE=1 SV=1		
	Gene.85733_N_geniculatus_18850_g.85733_m.85733	SLCB_DEIAC	Snaclec agkicetin-C subunit beta OS=Deinagkistrodon acutus OX=36307 PE=1 SV=1	4.1E-12	13.03
	Gene.50710_N_geniculatus_8234_g.50710_m.50710	SLA_DABSI	Snaclec dabocetin subunit alpha OS=Daboia siamensis OX=343250 PE=1 SV=1	4.5E-15	0.36
SNTX/VTX toxin family	Gene.184803_N_geniculatus_72767_g.184803_m.184803	VTXB_SYNVE	Verrucotoxin subunit beta OS=Synanceia verrucosa OX=51996 PE=1 SV=3	2.7E-22	0.00
	Gene.70470_N_geniculatus_13754_g.70470_m.70470	STXB_SYNHO	Stonustoxin subunit beta OS=Synanceia horrida OX=13279 PE=1 SV=3	1.3E-73	0.37
	Gene.83884_N_geniculatus_18187_g.83884_m.83884	CTXA_SCOPL	Cytolytic toxin-alpha OS=Scorpaena plumieri OX=274700 PE=1 SV=1	2.9E-74	0.72
	Gene.58615_N_geniculatus_10309_g.58615_m.58615	STXA_SYNHO	Stonustoxin subunit alpha OS=Synanceia horrida OX=13279 PE=1 SV=3	9.2E-89	0.78
	Gene.49719_N_geniculatus_7994_g.49719_m.49719	STXA_SYNVE	Neoverrucotoxin subunit alpha OS=Synanceia verrucosa OX=51996 PE=1 SV=1	5.4E-97	3.16
	Gene.16256_N_geniculatus_1578_g.16256_m.16256	STXB_SYNVE	Neoverrucotoxin subunit beta OS=Synanceia verrucosa OX=51996 PE=1 SV=1	1.9E-93	3.89
True venom lectin family	Gene.174251_N_geniculatus_65555_g.174251_m.174251	LECM_NOTSC	C-type lectin mannose-binding isoform OS=Notechis scutatus scutatus OX=70142 PE=2 SV=1	2.7E-14	0.78
Other toxins candidates					

Elevenin-like	Gene.89934_N_geniculatus_20379_g.89934_m.89934	CELE_CONVC	Elevenin-Vc1 OS=Conus victoriae OX=319920 PE=1 SV=1	5.5E-06	1.26
IGFBP N-terminal, Kazal-like, Ig-like C2-type	Gene.67877_N_geniculatus_12957_g.67877_m.67877	IGFBP_CUPSA	Insulin-like growth factor-binding protein-related protein 1 OS=Cupiennius salei OX=6928 PE=2 SV=1	4.4E-60	16.63
Insulin family	Gene.98850_N_geniculatus_23870_g.98850_m.98850	INS1_CONIM	Con-Ins Im1 OS=Conus imperialis OX=35631 PE=2 SV=1	3.2E-09	5.51
	Gene.86492_N_geniculatus_19114_g.86492_m.86492	INS2_CONIM	Con-Ins Im2 OS=Conus imperialis OX=35631 PE=2 SV=1	6.6E-15	1.98
	Gene.94322_N_geniculatus_22061_g.94322_m.94322	INS2C_CONFO	Con-Ins F2c OS=Conus floridulus OX=97180 PE=1 SV=1	6.1E-10	0.00
Glycoprotein hormones subunit alpha family	Gene.96349_N_geniculatus_22878_g.96349_m.96349	CTHA2_CONVC	Thyrostimulin alpha-2 subunit OS=Conus victoriae OX=319920 PE=1 SV=1	2.8E-17	2.97
Glycoprotein hormones subunit beta family	Gene.61031_N_geniculatus_10955_g.61031_m.61031	CTHB5_CONVC	Thyrostimulin beta-5 subunit OS=Conus victoriae OX=319920 PE=2 SV=1	1.3E-22	1.49
LDL-receptor class A domain	Gene.107185_N_geniculatus_27436_g.107185_m.107185	CPROH_CONVC	Neuropeptide prohormone-4 OS=Conus victoriae OX=319920 PE=1 SV=1	8E-25	5.63
NGF-beta family	Gene.108644_N_geniculatus_28085_g.108644_m.108644	NGFV_NAJAT	Venom nerve growth factor OS=Naja atra OX=8656 PE=1 SV=1	4.7E-10	0.23
	Gene.122061_N_geniculatus_34320_g.122061_m.122061	NGFV1_PSEAU	Venom nerve growth factor 1 OS=Pseudechis australis OX=8670 PE=2 SV=1	2.1E-10	0.19

	Gene.105448_N_geniculatus_26669_g.105448_m.105448	NGFV_DRYCN	Venom nerve growth factor OS=Drysdalia coronoides OX=66186 PE=2 SV=1	6.1E-13	3.83
	Gene.106335_N_geniculatus_27053_g.106335_m.106335	NGFV_PSEPO	Venom nerve growth factor OS=Pseudechis porphyriacus OX=8671 PE=2 SV=1	4.6E-14	0.00
	Gene.108412_N_geniculatus_27983_g.108412_m.108412	NGFV2_NAJSP	Venom nerve growth factor 2 OS=Naja sputatrix OX=33626 PE=2 SV=1	9.5E-18	0.29
PDGF/VEGF growth factor family	Gene.65140_N_geniculatus_12143_g.65140_m.65140	VEGFA_VIPAA	Vascular endothelial growth factor A OS=Vipera ammodytes ammodytes OX=8705 PE=2 SV=1	4.2E-09	3.28
Venom complement C3 homolog family	Gene.113391_N_geniculatus_30216_g.113391_m.113391	VCO3_NAJKA	Cobra venom factor OS=Naja kaouthia OX=8649 PE=1 SV=1	2.5E-24	0.48

Table S11. Toxin transcripts of *Riseriellus occultus_*.

Protein family	Transcript ID	Uniprot accession	Description	E-value	Expression, TPM
		Pore-forming toxins			
Cytotoxin A-III-like	Gene.68287_R_occultus_26947_g.68287_m.68287	CXA3_CERLA	Cytotoxin A-III OS=Cerebratulus lacteus OX=6221 PE=1 SV=1	1.7E-44	57.67
MACPF domain	Gene.49434_R_occultus_13168_g.49434_m.49434	PV21_POMMA	Perivitellin-2 67 kda subunit OS=Pomacea maculata OX=1245466 PE=1 SV=1	8.2E-59	8.34
		Enzymes			
Arthropod phospholipase D family. Class II subfamily	Gene.90642_R_occultus_56425_g.90642_m.90642	B1T1_LOXSN	Dermonecrotic toxin lspisictox-betaie3i (Fragment) OS=Loxosceles spinulosa OX=571532 PE=2 SV=1	1.1E-13	1.86

	Gene.72081_R_occultus_30849_g.72081_m.72081	B1O_LOXCS	Dermonecrotic toxin lcssictox-betaic1 (Fragment) OS=Loxosceles cf. Spinulosa (strain GJB-2008) OX=575952 PE=2 SV=1	2E-14	6.05
	Gene.75604_R_occultus_34760_g.75604_m.75604	A1MA4_LOXDE	Dermonecrotic toxin ldsictox-alphaib3aiv (Fragment) OS=Loxosceles deserta OX=424440 PE=2 SV=1	3.6E-16	1.53
	Gene.91645_R_occultus_58150_g.91645_m.91645	B1V_SICCD	Dermonecrotic toxin sdsictox-betaif1 (Fragment) OS=Sicarius cf. Damarensis (strain GJB-2008) OX=575956 PE=2 SV=1	1.4E-18	6.47
	Gene.56378_R_occultus_17338_g.56378_m.56378	DTPLD_HEMLE	Dermonecrotic toxin Hl-PLD1 OS=Hemiscorpius lepturus OX=520031 PE=1 SV=1	1.9E-22	7.64
Dnase II family	Gene.14621_R_occultus_1659_g.14621_m.14621	DNA2_ACAPL	Plancitoxin-1 OS=Acanthaster planci OX=133434 PE=1 SV=1	1.6E-98	3.51
Flavin monoamine oxidase family. FIG1 subfamily	Gene.28153_R_occultus_4706_g.28153_m.28153	OXLA_CALRH	L-amino-acid oxidase OS=Calloselasma rhodostoma OX=8717 PE=1 SV=2	2.7E-44	16.20
Natterin family	Gene.59007_R_occultus_19209_g.59007_m.59007	NATT4_THANI	Natterin-4 OS=Thalassophryne nattereri OX=289382 PE=2 SV=1	3.9E-14	7.80
Nucleotide pyrophosphatase/phosphodiesterase family	Gene.28090_R_occultus_4687_g.28090_m.28090	PDE2_CROAD	Venom phosphodiesterase 2 OS=Crotalus adamanteus OX=8729 PE=1 SV=1	8.9E-180	5.68

Phospholipase A2 family. Group I subfamily. D49 sub-subfamily	Gene.67220_R_occultus_25928_g.67220_m.67220	PA2TA_OXYSC	Basic phospholipase A2 taipoxin alpha chain OS=Oxyuranus scutellatus scutellatus OX=8667 PE=1 SV=1	6.3E-21	3.86
	Gene.34929_R_occultus_6835_g.34929_m.34929	PA2A2_NAJME	Acidic phospholipase A2 DE-II OS=Naja melanoleuca OX=8643 PE=1 SV=1	8.6E-25	40.35
Phospholipase A2 family. Group III subfamily	Gene.58712_R_occultus_19007_g.58712_m.58712	PA2_XYLAI	Phospholipase A(2) OS=Xylocopa appendiculata circumvolans OX=135722 GN=PLA2 PE=1 SV=1	9.9E-24	2.14
	Gene.13654_R_occultus_1496_g.13654_m.13654	PA2A2_HELSU	Acidic phospholipase A2 PA4 OS=Heloderma suspectum OX=8554 PE=1 SV=2	1.9E-32	3.84
Phospholipase B-like family	Gene.14123_R_occultus_1576_g.14123_m.14123	PLB_DRYCN	Phospholipase-B 81 OS=Drysdalia coronoides OX=66186 PE=1 SV=1	0	2.06
Venom metalloproteinase (M12B) family. P-I subfamily	Gene.88864_R_occultus_53388_g.88864_m.88864	VM1F_MACLB	Snake venom metalloproteinase fibrolase OS=Macrovipera lebetina OX=8709 PE=1 SV=1	1.2E-15	1.06
			Proteinase inhibitors		
Venom Kunitz-type family	Gene.52630_R_occultus_14975_g.52630_m.52630	VKT2_BITGA	Kunitz-type serine protease inhibitor bitisilin-2 OS=Bitis gabonica OX=8694 PE=1 SV=1	1.1E-15	32.42
	Gene.83158_R_occultus_44528_g.83158_m.83158	VKT_BOMTE	Kunitz-type serine protease inhibitor Bt-KTI OS=Bombus terrestris OX=30195 PE=2 SV=1	6.5E-17	327.70

	Gene.71074_R_occultus_29761_g.71074_m.71074	VKT3_BITGA	Kunitz-type serine protease inhibitor bitisilin-3 (Fragment) OS=Bitis gabonica OX=8694 PE=2 SV=1	3.5E-26	3.44
Venom Kunitz-type family. Sea anemone type 2 potassium channel toxin subfamily	Gene.46126_R_occultus_11483_g.46126_m.46126	ATPI1_ACTTE	Actinia tenebrosa protease inhibitors OS=Actinia tenebrosa OX=6105 PE=1 SV=1	9.8E-43	8.83
Ion channel inhibitor activity					
CRISP family	Gene.62190_R_occultus_21590_g.62190_m.62190	CRVP_HOPST	Cysteine-rich venom protein pseudechetoxin-like OS=Hoplocephalus stephensii OX=196418 PE=2 SV=1	8E-14	314.25
	Gene.51903_R_occultus_14556_g.51903_m.51903	TX31_CONTE	Cysteine-rich venom protein OS=Conus textile OX=6494 PE=1 SV=1	2.4E-26	331.75
	Gene.44413_R_occultus_10655_g.44413_m.44413	MR30_CONMR	Cysteine-rich venom protein Mr30 OS=Conus marmoreus OX=42752 PE=1 SV=1	4.1E-32	54.22
	Gene.49813_R_occultus_13368_g.49813_m.49813	CRVP_PSEAU	Cysteine-rich venom protein pseudechetoxin OS=Pseudechis australis OX=8670 PE=1 SV=1	1.9E-36	3.40
CRISP family. Venom allergen 5-like subfamily	Gene.59141_R_occultus_19305_g.59141_m.59141	VA543_SCODE	Scoloptoxin SSD43 OS=Scolopendra dehaani OX=2609776 PE=1 SV=1	6E-30	113.83
Nemertide family	Gene.67160_R_occultus_25870_g.67160_m.67160	NEMA1_LINLO	Nemertide alpha-1 (Fragment) OS=Lineus longissimus OX=88925 PE=1 SV=1	6.4E-12	2.02
Scoloptoxin-11 family	Gene.85657_R_occultus_48208_g.	TX14B_SCODE	Scoloptoxin SSD20 (Fragment) OS=Scolopendra dehaani	3.7E-36	2.62

	85657_m.85657		OX=2609776 PE=1 SV=1		
Venom Kunitz-type family. Sea anemone type 2 potassium channel toxin subfamily	Gene.45107_R_occultus_10986_g.45107_m.45107	VKT53_ANEVI	Kappapi-actitoxin-Avd3e OS=Anemonia viridis OX=51769 PE=3 SV=1	4.1E-18	168.01
Hemostasis impairing toxin					
Snaclec family	Gene.68472_R_occultus_27105_g.68472_m.68472	SL9A_CROAD	C-type lectin 9a OS=Crotalus adamanteus OX=8729 PE=1 SV=1	1.4E-08	89.97
	Gene.38071_R_occultus_7965_g.38071_m.38071	SLBB_TRIAB	Snaclec alboaggregin-B subunit beta OS=Trimeresurus albolabris OX=8765 PE=1 SV=2	2.5E-08	6.70
SNTX/VTX toxin family	Gene.24886_R_occultus_3814_g.24886_m.24886	STXA_SYNVE	Neoverrucotoxin subunit alpha OS=Synanceia verrucosa OX=51996 PE=1 SV=1	4.9E-42	5.02
	Gene.45481_R_occultus_11159_g.45481_m.45481	STXB_SYNVE	Neoverrucotoxin subunit beta OS=Synanceia verrucosa OX=51996 PE=1 SV=1	3.5E-78	4.53
	Gene.911_R_occultus_40_g.911_m.911	STXA_SYNHO	Stonustoxin subunit alpha OS=Synanceia horrida OX=13279 PE=1 SV=3	8.5E-88	6.48
	Gene.7321_R_occultus_590_g.7321_m.7321	CTXA_SCOPL	Cytolytic toxin-alpha OS=Scorpaena plumieri OX=274700 PE=1 SV=1	6.8E-95	4.72
True venom lectin family	Gene.91840_R_occultus_58524_g.91840_m.91840	LECG_THANI	Galactose-specific lectin nattectin OS=Thalassophryne nattereri OX=289382 PE=1 SV=1	4.5E-10	3.12
Other toxins candidates					
IGFBP N-terminal domain	Gene.96120_R_occultus_66363_g.	VP302_LYCMC	Venom protein 302 OS=Lychas mucronatus OX=172552 PE=2 SV=1	2E-06	1.61

	96120_m.96120				
IGFBP N-terminal, Kazal-like, Ig-like C2-type	Gene.24185_R_occultus_3642_g.24185_m.24185	IGFBP_CUPSA	Insulin-like growth factor-binding protein-related protein 1 OS=Cupiennius salei OX=6928 PE=2 SV=1	1.8E-52	7.69
Insulin family	Gene.89464_R_occultus_54345_g.89464_m.89464	INS1_CONIM	Con-Ins Im1 OS=Conus imperialis OX=35631 PE=2 SV=1	7.8E-07	5.56
	Gene.47239_R_occultus_12039_g.47239_m.47239	INS1_CONFO	Con-Ins F1 OS=Conus floridulus OX=97180 PE=1 SV=1	9.9E-10	7.39
LDL-receptor class A domain	Gene.38355_R_occultus_8068_g.38355_m.38355	CPROH_CONVC	Neuropeptide prohormone-4 OS=Conus victoriae OX=319920 PE=1 SV=1	1.5E-24	28.02
-SE-CTX-like	Gene.14754_R_occultus_1682_g.14754_m.14754	CTXL_ACRMI	Cephalotoxin-like protein (Fragment) OS=Acropora millepora OX=45264 PE=1 SV=1	3.7E-144	46.79
Vasopressin/oxytocin family	Gene.65983_R_occultus_24746_g.65983_m.65983	CESS2_CONMO	Conopressin/conophysin, isoform 2 (Fragment) OS=Conus monile OX=351660 PE=2 SV=1	4.3E-24	3.06

Table S12. Toxin transcripts of *Amphiporous lactifloreus*.

Protein family	Transcript ID	Uniprot accession	Description	E-value	Expression, TPM
		Pore-forming toxins			
MACPF domain	Gene.38401_Amphiporous_6886_g.38401_m.38401	PV21_POMCA	Perivitellin-2 67 kda subunit OS=Pomacea canaliculata OX=400727 PE=1 SV=2	8.7E-39	2.49
	Gene.31194_Amphiporous_4943_g.31194_m.31194	PV21_POMMA	Perivitellin-2 67 kda subunit OS=Pomacea maculata OX=1245466 PE=1 SV=1	1.2E-73	435.18

Enzymes					
Dnase II family	Gene.56234_Amphiporous_13892_g.56234_m.56234	DNA2_ACAPL	Plancitoxin-1 OS=Acanthaster planci OX=133434 PE=1 SV=1	3.6E-36	2.00
Glycosyl hydrolase 56 family	Gene.65205_Amphiporous_20146_g.65205_m.65205	HUGA_APIME	Hyaluronidase OS=Apis mellifera OX=7460 PE=1 SV=1	8.9E-14	3.13
	Gene.65057_Amphiporous_20037_g.65057_m.65057	HYAL_CONPU	Hyaluronidase conohyal-P1 OS=Conus purpurascens OX=41690 PE=1 SV=1	5.8E-12	2.47
	Gene.34524_Amphiporous_5791_g.34524_m.34524	HYAL1_MESMA	Hyaluronidase-1 OS=Mesobuthus martensii OX=34649 PE=1 SV=2	2.4E-59	5.82
**Peptidase M12A domain, Allrgn_V5/Tpx1_CS, CAP_domain**	**Gene.50662_Amphiporous_11207_g.50662_m.50662**	**VMPA2_LOXIN**	**Astacin-like metalloprotease toxin 2 OS=Loxosceles intermedia OX=58218 PE=2 SV=1**	**5.2E-32**	**260.11**
Phospholipase A2 family. Group III subfamily	Gene.64909_Amphiporous_19895_g.64909_m.64909	PA23_HELSU	Phospholipase A2 isozymes PA3A/PA3B/PA5 OS=Heloderma suspectum OX=8554 PE=1 SV=3	2E-19	1029.82
Type-B carboxylesterase/lipase family	Gene.23960_Amphiporous_3328_g.23960_m.23960	ACES_BUNFA	Acetylcholinesterase OS=Bungarus fasciatus OX=8613 GN=ACHE PE=1 SV=2	5.3E-126	15.33
Proteinase inhibitors					
Venom Kunitz-type family	Gene.41216_Amphiporous_7730_g.41216_m.41216	VKT1D_CONCL	Kunitz-type serine protease inhibitor conotoxin Cal9.1d (Fragment) OS=Californiconus californicus OX=1736779 PE=2 SV=1	1.1E-16	35.41

Venom Kunitz-type family. 02 (native) subfamily	Gene.52236_Amphiporous_11892_g.52236_m.52236	VKT1_ARAVE	Kunitz-type U1-aranetoxin-Av1a OS=Araneus ventricosus OX=182803 PE=2 SV=1	5.6E-8	10.78
Venom Kunitz-type family. Sea anemone type 2 potassium channel toxin subfamily	Gene.27479_Amphiporous_4081_g.27479_m.27479	VKT3C_ACTEQ	PI-actitoxin-Aeq3c OS=Actinia equina OX=6106 PE=1 SV=1	7.8E-14	129.20
Ion channel inhibitor activity					
CRISP family. Venom allergen 5-like subfamily	Gene.59180_Amphiporous_15642_g.59180_m.59180	VA552_SCODE	Scoloptoxin SSD552 OS=Scolopendra dehaani OX=2609776 PE=1 SV=1	7.3E-17	3.27
	Gene.49571_Amphiporous_10741_g.49571_m.49571	VA576_SCODE	Scoloptoxin SSD976 OS=Scolopendra dehaani OX=2609776 PE=1 SV=1	6.7E-23	30.43
Sea anemone structural class 9a family	Gene.21587_Amphiporous_2850_g.21587_m.21587	TX9A_ANTMC	Delta-actitoxin-Amc1a OS=Antheopsis maculata OX=280228 PE=1 SV=1	1.8E-6	114.61
Scoloptoxin-11 family	Gene.78441_Amphiporous_37091_g.78441_m.78441	TX14B_SCODE	Scoloptoxin SSD20 (Fragment) OS=Scolopendra dehaani OX=2609776 PE=1 SV=1	2E-10	2.80
Hemostasis impairing toxin					
Snaclec family	Gene.59882_Amphiporous_16109_g.59882_m.59882	SL9A_PROFL	Snaclec coagulation factor IX/factor X-binding protein subunit A OS=Protobothrops flavoviridis OX=88087 PE=1 SV=2	4.8E-6	1.89
	Gene.59450_Amphiporous_15814_g.59450_m.59450	SLA_BOTIN	Snaclec bothroinsularin subunit alpha OS=Bothrops insularis OX=8723 PE=1 SV=1	2E-6	1.91
	Gene.60327_Amphiporous_16401_g.60327_m.60327	SLA_GLOBR	Snaclec salmorin subunit A OS=Gloydius brevicaudus	4.9E-8	243.07

			OX=259325 PE=1 SV=1		
	Gene.81843_ Amphiporous_42700_ g.81843_m.81 843	SLA1_DEIAC	Snaclec agglucetin subunit alpha-1 OS=Deinagkistrodon acutus OX=36307 PE=1 SV=1	1E-6	2.03
	Gene.58164_ Amphiporous_15029_ g.58164_m.58 164	SLRB_BITRH	Snaclec rhinocetin subunit beta OS=Bitis rhinoceros OX=715877 PE=1 SV=1	5.1E-6	4.70
	Gene.66006_ Amphiporous_20850_ g.66006_m.66 006	SLA_BITAR	Snaclec bitiscetin subunit alpha OS=Bitis arietans OX=8692 PE=1 SV=1	2.4E-6	3.02
True venom lectin family	Gene.66666_ Amphiporous_21506_ g.66666_m.66 666	LECM1_ERYPO	C-type lectin lectoxin-Lio1 OS=Erythrolamprus poecilogyrus OX=338838 PE=2 SV=1	2.8E-09	2.49
	Gene.63849_ Amphiporous_18988_ g.63849_m.63 849	LECM1_HYDHA	C-type lectin 1 OS=Hydrophis hardwickii OX=8781 PE=2 SV=1	2.7E-07	1.82
Other toxins candidates					
Insulin family	Gene.56064_ Amphiporous_13794_ g.56064_m.56 064	INS1_CONTE	Con-Ins Tx1 OS=Conus textile OX=6494 PE=2 SV=1	6.8E-06	2.61
**Insulin-like growth factor-binding protein domain, Kazal-type serine protease inhibitor domain, Immunoglobulin domain**	**Gene.38477_ Amphiporous_6906_g. 38477_m.384 77**	**IGFBP_CUPSA**	**Insulin-like growth factor-binding protein-related protein 1 OS=Cupiennius salei OX=6928 PE=2 SV=1**	**2.5E-46**	**67.34**
LDL-receptor class A domain	Gene.44369_ Amphiporous_8766_g.	CPROH_CONVC	Neuropeptide prohormone-4 OS=Conus victoriae	2E-19	24.69

	44369_m.44369		OX=319920 PE=1 SV=1		
NGF-beta family	Gene.54049_Amphiporous_12770_g.54049_m.54049	NGFV_PSEPO	Venom nerve growth factor OS=Pseudechis porphyriacus OX=8671 PE=2 SV=1	3.4E-12	12.89
PDGF/VEGF growth factor family	Gene.37959_Amphiporous_6754_g.37959_m.37959	VEGFA_VIPAA	Vascular endothelial growth factor A OS=Vipera ammodytes ammodytes OX=8705 PE=2 SV=1	2.1E-10	7.12
TCTP family	Gene.54532_Amphiporous_13003_g.54532_m.54532	TCTP_BRABE	Translationally-controlled tumor protein homolog OS=Branchiostoma belcheri OX=7741 PE=2 SV=1	3.5E-27	2253.54

Table S13. Toxin transcripts of *Malacobdella grossa*.

**Protein family**	**Transcript ID**	**Uniprot accession**	**Description**	**E-value**	**Expression, TPM**
			Pore-forming toxins		
MACPF domain	Gene.21779_M_grossa_5772_g.21779_m.21779	PV21_POMMA	Perivitellin-2 67 kda subunit OS=Pomacea maculata OX=1245466 PE=1 SV=1	5.6E-59	209.15
	Gene.37448_M_grossa_16878_g.37448_m.37448	PV21_POMCA	Perivitellin-2 67 kda subunit OS=Pomacea canaliculata OX=400727 PE=1 SV=2	1.9E-11	15.35
			Enzymes		
Dnase II family	Gene.18803_M_grossa_4604_g.18803_m.18803	DNA2_ACAPL	Plancitoxin-1 OS=Acanthaster planci OX=133434 PE=1 SV=1	3.8E-74	13.52
Glycosyl hydrolase 56 family	Gene.25244_M_grossa_7377_g.25244_m.25244	HYAL1_MESMA	Hyaluronidase-1 OS=Mesobuthus martensii OX=34649 PE=1 SV=2	5.3E-70	5.02
Phospholipase A2 family. Group III subfamily	Gene.32339_M_grossa_11758_g.32339_m.32339	PA23_HELSU	Phospholipase A2 isozymes PA3A/PA3B/PA5 OS=Heloderma	1.7E-18	57.55

			suspectum OX=8554 PE=1 SV=3		
Proteinase inhibitors					
Venom Kunitz-type family	Gene.26006_M_grossa_7764_g.26006_m.26006	VKT1_OXYMI	Kunitz-type serine protease inhibitor microlepidin-1 OS=Oxyuranus microlepidotus OX=111177 PE=2 SV=1	4.8E-11	64.37
	Gene.31449_M_grossa_11069_g.31449_m.31449	VKT_PSEPC	Kunitz-type serine protease inhibitor PPTI OS=Pseudocerastes persicus OX=47769 PE=1 SV=1	1.4E-14	10.66
Venom Kunitz-type family. 02 (native) subfamily	Gene.33811_M_grossa_13000_g.33811_m.33811	VKT17_CYRHA	Kunitz-type serine protease inhibitor HNTX-03141017 (Fragment) OS=Cyriopagopus hainanus OX=209901 PE=2 SV=1	4.3E-12	8.67
Ion channel inhibitor activity					
CRISP family	Gene.14471_M_grossa_3136_g.14471_m.14471	CRVP_LATSE	Cysteine-rich venom protein latisemin OS=Laticauda semifasciata OX=8631 PE=2 SV=1	4.8E-23	5.94
CRISP family. Venom allergen 5-like subfamily	Gene.20111_M_grossa_5118_g.20111_m.20111	VA576_SCODE	Scoloptoxin SSD976 OS=Scolopendra dehaani OX=2609776 PE=1 SV=1	2.3E-26	5.64
Sea anemone structural class 9a family	Gene.29516_M_grossa_9755_g.29516_m.29516	TX9A_ANTMC	Delta-actitoxin-Amc1a OS=Antheopsis maculata OX=280228 PE=1 SV=1	1.7E-14	174.77
Hemostasis impairing toxin					
SNTX/VTX toxin family	Gene.20782_M_grossa_5372_g.20782_m.20782	VTXB_SYNVE	Verrucotoxin subunit beta OS=Synanceia verrucosa OX=51996 PE=1 SV=3	3.9E-06	6.41
True venom lectin family	Gene.31520_M_grossa_11	LECM4_PSEPL	C-type lectin lectoxin-Enh4 OS=Pseudoferania	6E-08	12.83

	121_g.31520_ m.31520		polylepis OX=338839 PE=2 SV=1		
		Other toxins candidates			
IGFBP N-terminal, Kazal-like, Ig-like C2-type	Gene.23069_ M_grossa_6346_g.23069_ m.23069	IGFBP_CUPSA	Insulin-like growth factor-binding protein-related protein 1 OS=Cupiennius salei OX=6928 PE=2 SV=1	2E-47	8.91
LDL-receptor class A domain	Gene.30471_ M_grossa_10361_g.30471_ m.30471	CPROH_CONVC	Neuropeptide prohormone-4 OS=Conus victoriae OX=319920 PE=1 SV=1	2.9E-21	26.05
PDGF/VEGF growth factor family	Gene.21287_ M_grossa_5568_g.21287_ m.21287	VEGFA_VIPAA	Vascular endothelial growth factor A OS=Vipera ammodytes ammodytes OX=8705 PE=2 SV=1	6.7E-11	4.89

Table S14. Toxin transcripts of *Paranemertes peregrina*. Unique toxins families are shown in bold.

Protein family	Transcript ID	Uniprot accession	Description	E-value	Expression, TPM
		Enzymes			
Glycosyl hydrolase 56 family	Gene.13505_ P_peregrina_2885_g .13505_m.13505	HYAL1_MESMA	Hyaluronidase-1 OS=Mesobuthus martensii OX=34649 PE=1 SV=2	1.6E-59	4.15
**Venom metalloproteinase (M12B) family. P-II subfamily. P-iia sub-subfamily**	**Gene.40257_ P_peregrina_19132_ g.40257_m.40257**	**VM2RH_CALRH**	**Zinc metalloproteinase/disintegrin OS=Calloselasma rhodostoma OX=8717 PE=1 SV=2**	**3.3E-16**	**4.6**
		Proteinase inhibitors			
Venom Kunitz-type family	Gene.34086_ P_peregrina_13107_ g.34086_m.34086	VKT1D_CONCL	Kunitz-type serine protease inhibitor conotoxin Cal9.1d (Fragment) OS=Californiconus californicus OX=1736779 PE=2 SV=1	7.9E-15	9.22
Venom Kunitz-type family. Sea anemone type 2	Gene.30860_ P_peregrina_10693_	VKT3_ANTAF	PI-actitoxin-Axm2b OS=Anthopleura aff.	1.2E-15	110.86

potassium channel toxin subfamily	g.30860_m.30860		Xanthogrammica OX=152178 PE=1 SV=1		
Ion channel inhibitor activity					
CRISP family. Venom allergen 5-like subfamily	Gene.6371_P_peregrina_1018_g.6371_m.6371	VA576_SCODE	Scoloptoxin SSD976 OS=Scolopendra dehaani OX=2609776 PE=1 SV=1	1.1E-28	11.64
Scoloptoxin-11 family	Gene.23106_P_peregrina_6437_g.23106_m.23106	TX14B_SCODE	Scoloptoxin SSD20 (Fragment) OS=Scolopendra dehaani OX=2609776 PE=1 SV=1	2.2E-20	4.00
Hemostasis impairing toxin					
Snaclec family	Gene.34963_P_peregrina_13832_g.34963_m.34963	SLAE_MACLB	Snaclec A14 OS=Macrovipera lebetina OX=8709 PE=2 SV=1	3.5E-6	3.00
	Gene.38662_P_peregrina_17339_g.38662_m.38662	SLLC2_DABSI	Snaclec coagulation factor X-activating enzyme light chain 2 OS=Daboia siamensis OX=343250 GN=LC2 PE=1 SV=1	3.8E-10	8.49
SNTX/VTX toxin family	Gene.2895_P_peregrina_341_g.2895_m.2895	STXA_SYNVE	Neoverrucotoxin subunit alpha OS=Synanceia verrucosa OX=51996 PE=1 SV=1	3.7E-13	14.73
	Gene.14425_P_peregrina_3163_g.14425_m.14425	CTXA_SCOPL	Cytolytic toxin-alpha OS=Scorpaena plumieri OX=274700 PE=1 SV=1	8.7E-14	8.72
Other toxins candidates					
LDL-receptor class A domain	Gene.17193_P_peregrina_4084_g.17193_m.17193	CPROH_CONVC	Neuropeptide prohormone-4 OS=Conus victoriae OX=319920 PE=1 SV=1	2.5E-19	8.15
Venom complement C3 homolog family	Gene.8194_P_peregrina_1422_g.8194_m.8194	VCO31_AUSSU	A.superbus venom factor 1 OS=Austrelaps superbus OX=29156 PE=1 SV=1	5.2E-49	9.40
